# Supplementary material for: Drought-Induced Civil Conflict Among the Ancient Maya
Source: Nat Commun. 2022 Jul 19;13:3911. doi: 10.1038/s41467-022-31522-x (PMC9296624; doi:10.1038/s41467-022-31522-x)
Supplement: Supplementary file 1 — Supplementary Information [file 41467_2022_31522_MOESM1_ESM.pdf]

Supplementary information

Drought-Induced Civil Conflict Among the Maya

In the format provided by the authors and unedited

Douglas J. Kennett*, Marilyn Masson, Carlos Peraza Lope, Stanley Serafin, Richard J. George, Tom C. Spencer, Julie A. Hoggarth, Brendan J. Culleton, Thomas K. Harper, Keith M. Prufer, Susan Milbrath, Bradley W. Russell, Eunice Uc González, Weston C. McCool, Valorie V. Aquino, Elizabeth H. Paris, Jason H. Curtis, Norbert Marwan, Mingua Zhang, Yemane Asmerom, Victor J. Polyak, Stacy A. Carolin, Daniel H. James, Andrew J. Mason, Gideon M. Henderson, Mark Brenner, James U.L. Baldini, Sebastian F.M. Breitenbach, David A. Hodell*

*corresponding authors

CONTENTS

Supplementary Note 1 (pp. 3–5):

SPELEOTHEM-BASED PALEOCLIMATE RECONSTRUCTIONS

Supplementary Figure 1 3

Supplementary Figure 2 5

Supplementary Note 2 (pp. 6–13):

LOCAL EVIDENCE FOR DROUGHT AT MAYAPAN: 1350-1450 CE

Supplementary Figure 3 6

Supplementary Figure 4 7

Supplementary Figure 5 8

Supplementary Figure 6 9

Supplementary Table 1 10

Supplementary Figure 7 12

Supplementary Figure 8 13

Supplementary Note 3 (pp. 14–26):

BURIAL CONTEXTS

DISTINGUISHING VIOLENT DEATHS FROM PROPER BURIALS

Supplementary Figure 9 15

Supplementary Figure 10 16

Supplementary Figure 11 18

Supplementary Figure 12 19

Supplementary Figure 13 20

Supplementary Note 4 (pp. 26–30):

STATISTICAL MODELS

SUMMED PROBABILITY DISTRIBUTIONS

Supplementary Table 2 28

Supplementary Figure 14 28

Supplementary Figure 15 29

Supplementary Figure 16 30

Supplementary References (pp. 31–35)

Supplementary Note 1:
SPELEOTHEM-BASED PALEOCLIMATE RECONSTRUCTIONS

The YOK-I speleothem stable isotope record ($\delta^{13}C$ and $\delta^{18}O$) from southern Belize was published previously and details are found in refs.[1–3]. For this study, we compared the original YOK-I record with the $\delta^{18}O$ record from the Chaac stalagmite from Tzabnah Cave, Tecoh, northern Yucatán (Supplementary Figure 1)[4]. The average chronological uncertainty for the latter

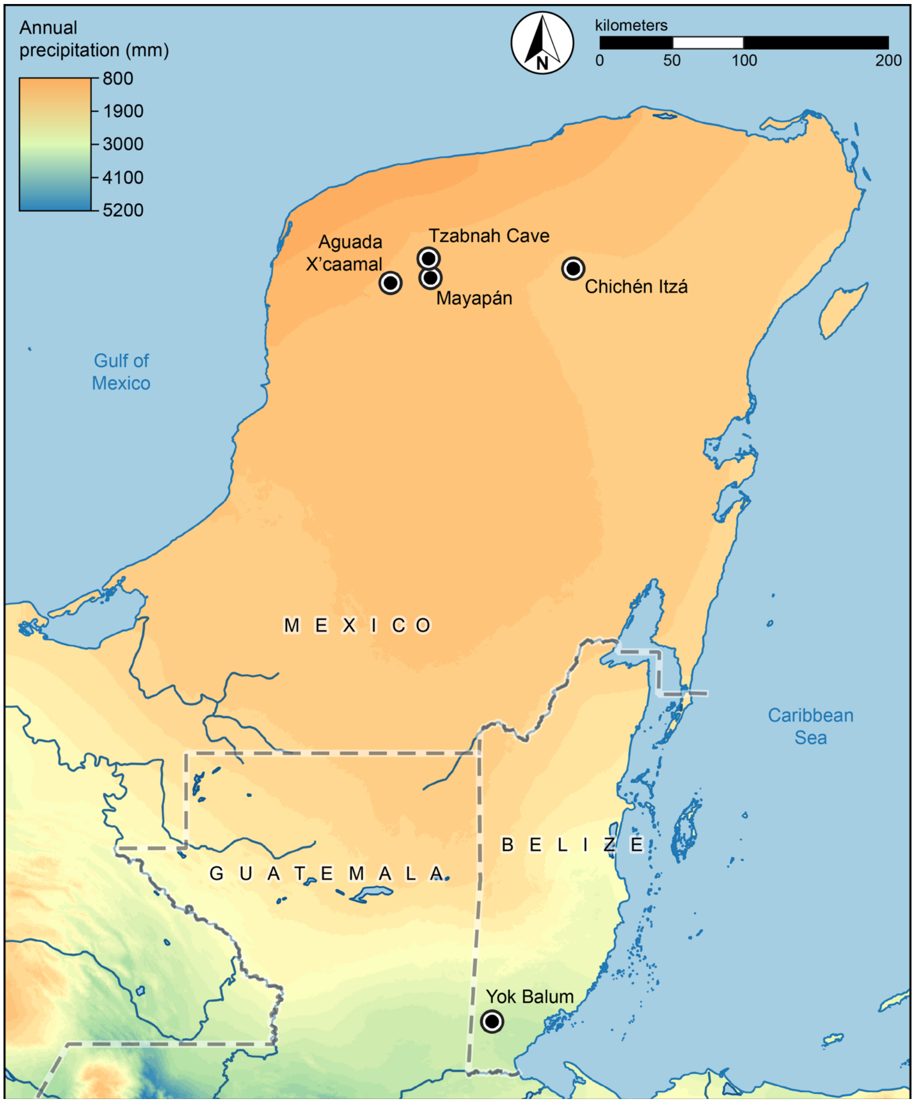

Supplementary Figure 1 | Map showing archaeological sites and climate archives relative to the regional rainfall gradient. Rainfall in the northwestern Yucatán Peninsula is marked by a strong north-south gradient. Distribution shown relative to major archaeological sites and paleoclimate archives.

record is ±70 years, which means that multi-decadal variations in climate conditions at the site could not be determined with confidence. Despite the chronological uncertainties, we found that between 1330 and 1430 CE, the average $\delta^{18}O$ value in the Chaac stalagmite from Tzabnah Cave shifted to -4.9‰, which is a higher multidecadal mean compared to the average $\delta^{18}O$ values from periods preceding and following (-5.3‰), 1100-1330 CE and 1430-1550 CE, suggesting drier conditions in northern Yucatán after ca. 1330 CE. Southern Belize also experienced drier conditions, as reflected by higher $\delta^{18}O$ values in both the YOK-I and YOK-G stalagmites.

To test if these spatially distant records were similar statistically, we compared them using kernel-based correlation analysis provided by the NESToolbox[5]. Whereas droughts might not affect the Yucatán Peninsula uniformly, we used this test to identify possible larger-scale atmospheric linkages. If such teleconnections do indeed affect the entire peninsula, we would expect to find similar climate-proxy relationships, with more negative $\delta^{18}O$ and lower $\delta^{13}C$ values in the speleothems associated with times of greater rainfall. Under this assumption, we matched the YOK-I and Tzabnah paleoclimate records with the YOK-G stalagmite from Southern Belize[6], within their 2σ error margins. Age models were developed using the COPRA routine with 1000 Monte Carlo simulations to obtain the 95% confidence limits[7]. For the matching procedure and correlation, the median YOK-G $\delta^{13}C$, YOK-I $\delta^{18}O$, and Tzabnah $\delta^{18}O$ time series were low-pass-filtered with Gaussian kernel smoothing at a bandwidth of 25 years, using NESToolbox[5]. This procedure removed the long-term variation in the records and highlighted shorter (sub-decadal) variability. Resulting adjusted chronologies are shown in Supplementary Figure 2, and the matched Tzabnah record is found in Supplementary Table 1. Given the temporally and spatially variable impact of atmospheric features (*nortes*, trades, storm tracks, etc.) across the Yucatán Peninsula, a perfect match among all these records was not expected. Future work involving high-resolution climate reconstructions from multiple sites around the region should be directed at validating the links suggested in this analysis. Paleoclimate networks would enable us to evaluate the spatial extent, leads, and lags of climate features, including droughts.

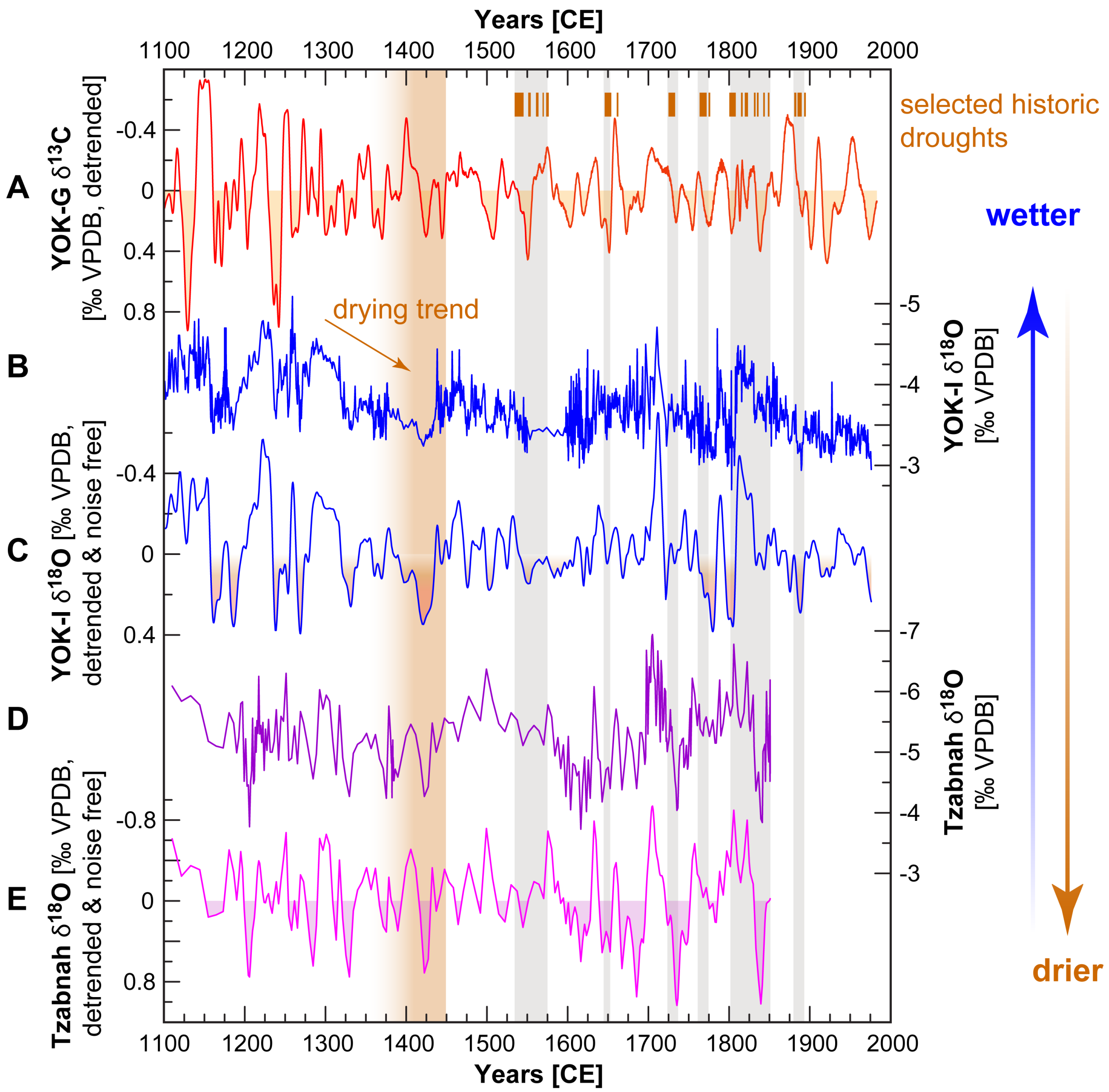

Supplementary Figure 2 | Speleothem-based proxy records and historical droughts in the Yucatán Peninsula. a The detrended $\delta^{13}C$ record from stalagmite YOK-G reflects local hydrology[6]. **b, c** The original and detrended $\delta^{18}O$ record from stalagmite YOK-I[1] shows the drying trend of the 14th century (red shading) and multiple multi-annual droughts. **d, e** The original and the detrended and noise-filtered $\delta^{18}O$ profile from Tzabnah Cave[4] exhibits similar variability and drought events. The original Tzabnah Cave and YOK-I isotope records have been matched within dating uncertainties to the YOK-G record, which has a superior chronology. Historic droughts[8] are shown for comparison (brown squares and grey shading).

Supplementary Note 2:
LOCAL EVIDENCE FOR DROUGHT AT MAYAPAN: 1350-1450 CE

Two other local paleoclimate records support our claim that a series of extended droughts occurred at Mayapan.

Local speleological evidence
In 2005, stalagmite M1 was retrieved from a karst cave located directly below the central plaza at Mayapan and accessible via Cenote Ch'en Mul (Supplementary Figure 3a and 3b). The cave is oriented in a SW-NE direction (Supplementary Figure 3c) and terminates in a sump with standing water. The cave was used for ceremonial purposes and served as a source of freshwater during the time of occupation of Mayapan[9,10].

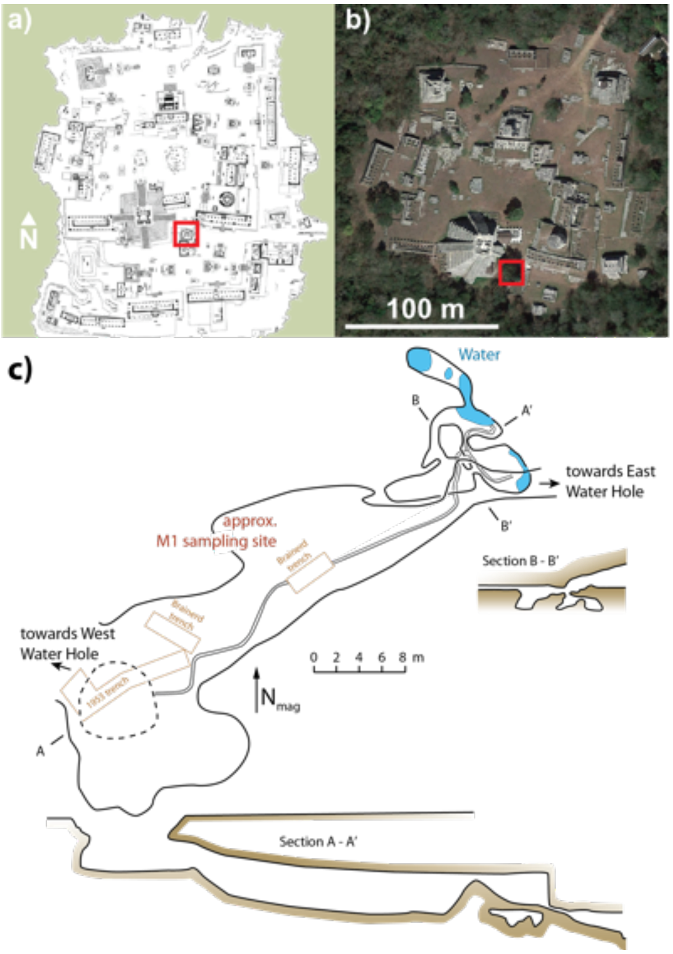

Supplementary Figure 3 | Location of the the Ch'en Mul cenote and stalagmite M1. a, b Map view and satellite image of the central precinct at Mayapan **a, b** The red square indicates the location of Ch'en Mul cenote. **c** Plan and cross sections of the cave*[11]*.

Stalagmite M1 was collected in the main chamber, approximately 20 m from the cave entrance. It is ~110 mm tall and ranges in width from about 40 to 65 mm. Petrographic evidence reveals two prominent detrital layers at ~28.0 mm and 26.6 mm from the top (Supplementary Figure 4), with very dark calcite deposition between both layers.

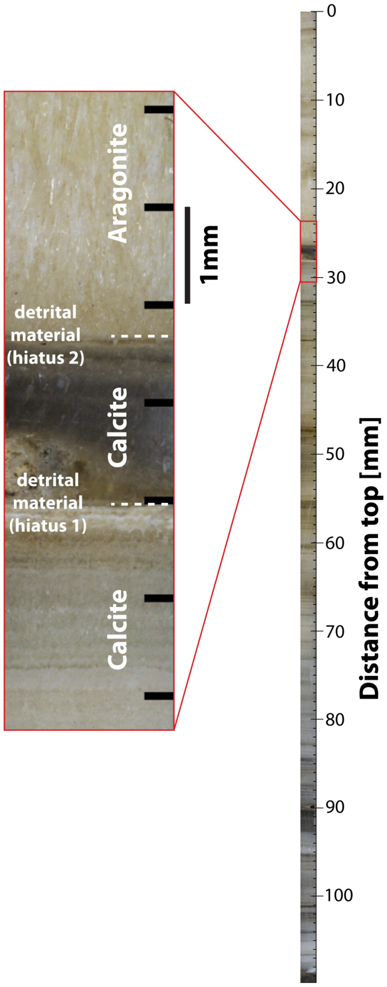

Supplementary Figure 4 | Stalagmite M1 profile. Petrographic evidence suggests two hiatuses, characterized by greater abundance of detrital material, which bracket the dark calcite layer between 28.0 and 26.6 mm from the top. Current radiometric dating precludes estimating the age of the dark layer, and distinction of proposed hiatuses 1 and 2.

The stubby stalagmite shows a well-developed horizontal apex. Fluorescent imaging, using a Leica SP8 inverted confocal microscope at the Cambridge Advanced Imaging Centre, shows clear laminations in the calcite section of M1 below 28.0 mm distance from the top (Supplementary Figure 5). These laminations are interpreted as annual layers, given the local intensely seasonal rainfall pattern[12].

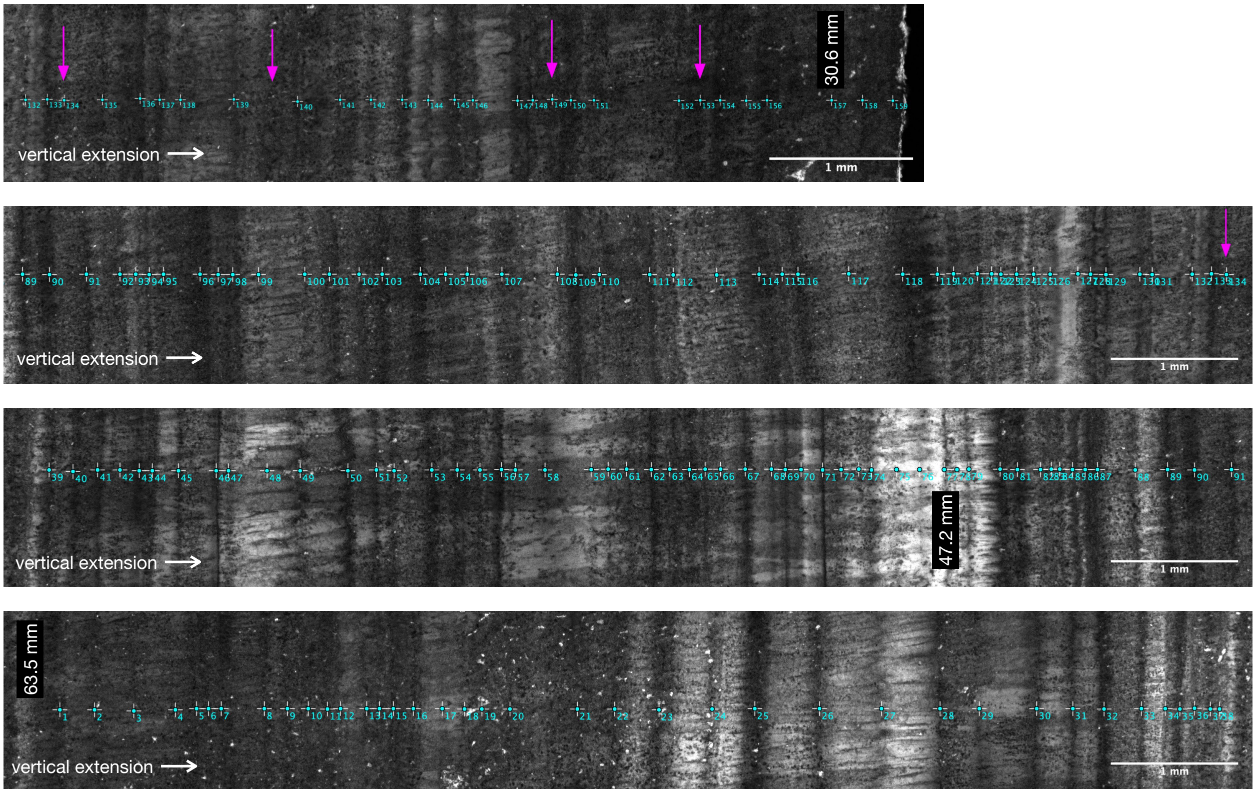

Supplementary Figure 5 | Fluorescence image of lower calcite section of stalagmite M1 with layer counts. U-Th age locations are indicated with 63.5 mm, 47.2 mm, and 30.6 mm labels. Layer numbers increase from older to younger along the stalagmite growth axis. The layer counting has been repeated independently four times by two people (SFMB and SAC) counting twice each before comparing numbers. Purple arrows indicate questionable growth layers.

Below 26.6 mm, M1 is composed of calcite interspersed with distinct detrital layers, whereas above the dark calcite layer, only clean, whitish/yellow aragonite was deposited. X-ray diffraction analysis at Cambridge confirmed that this change in character reflects a change in mineralogy: the lower section, including the ~1.2-mm-thick dark layer, is calcitic, whereas the top section is composed of aragonite. This is corroborated by a change in Sr/Ca values, measured by μ-XRF, which are significantly greater in the more recent section (Supplementary Figure 6[13]). Carbonate samples from the M1 speleothem were spiked with a mixture of ^{229}Th-^{236}U dissolved in HNO_3 and then refluxed on a hot-plate overnight for spike-sample equilibration. Chemical separation of U and Th from the sample matrix follows procedures adapted from[14]. Measurement of U

and Th was performed by Nu-Instrument Multicollector-Inductively-Coupled-Plasma-Mass Spectrometer (MC-ICP-MS) at the University of Oxford.

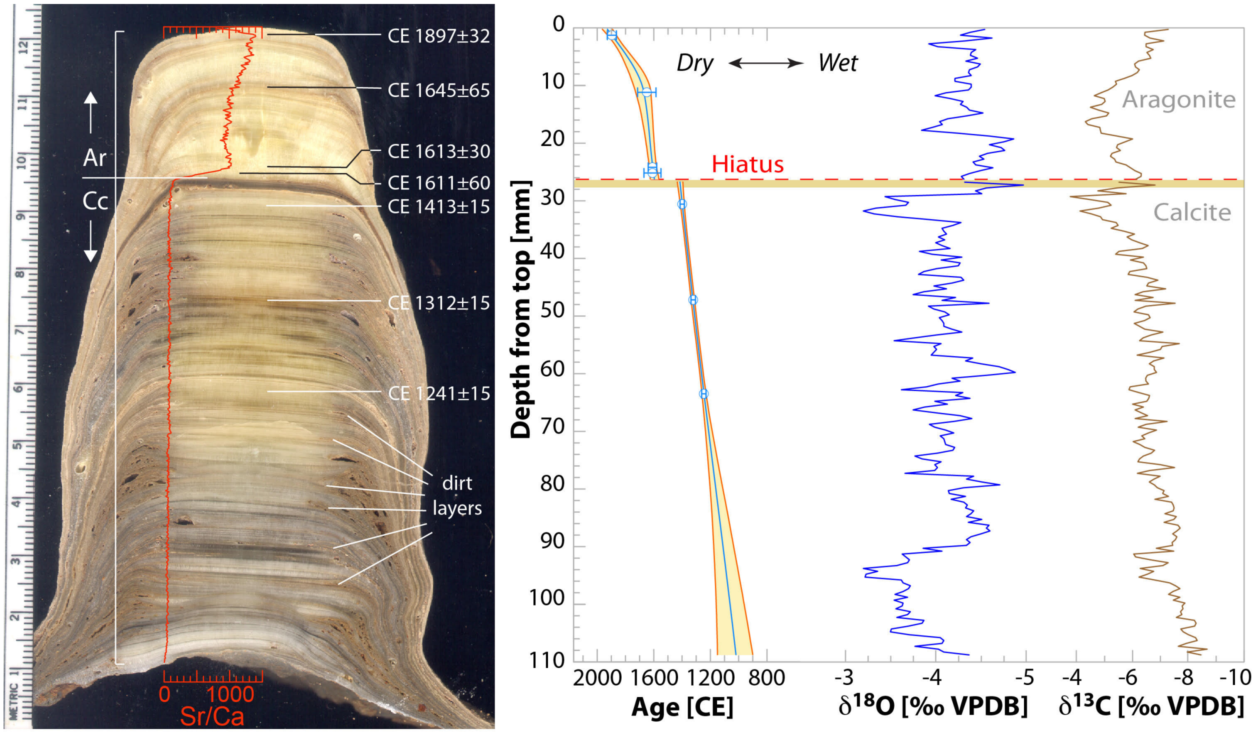

Supplementary Figure 6 | Image of stalagmite M1 with U-Th dates and Sr/Ca records superimposed (left), and its age model and stable isotope profiles (right). The increase in Sr/Ca at ca. 27±0.5 mm reflects a mineralogical change from calcite to aragonite that occurs above the hiatus, which spans the period from ca. 1410-1600 CE. M1 shows frequent dirt layers below the hiatus interval, which is discernible at 26.6 mm from the top, just above a very dark calcite layer (see Supplementary Figure 4 for close-up), above which there is an abrupt mineralogical transition from calcite to aragonite. Above this distinct hiatus, flood-induced clay deposition all but disappears. All data provided in Source Data File.

An age model was constructed for the lower calcite portion of the stalagmite (from its base to 28.0 mm from the top) by combining the constraints provided by U-Th dated samples and annual layer counting. Three “clean” (low detrital contamination) U-Th ages are located in the lower calcite section of M1 at 63.5 mm, 47.2 mm, and 30.6 mm from the top (Supplementary Figures 5 and 6), with the cleanest most precise U-Th age at 63.5 mm. Beginning at the 63.5 mm U-Th sample and counting annual layers upwards in the stalagmite, the level of the 47.2 mm U-Th date is aligned with the 77th layer (77 years younger than the sample at 63.5 mm), and the 30.6 mm U-Th level aligns with the 151st-156th layer (151-156 years younger than the sample at 63.5 mm), due to 5 layers being questionable (Supplementary Figure 5). Accuracy of the U-Th ages is dependent on the accuracy of the chosen initial (at the time of stalagmite deposition) $^{230}Th/^{232}Th$ ratio, used to correct for detrital Th contamination in the U-Th sample. We tested a large range of possible $^{230}Th/^{232}Th$ ratio values used to correct U-

Th ages for initial thorium contamination and found that the median $^{230}Th/^{232}Th$ ratio that simulates the most solutions consistent with the layer count constraints was 117 ppm, with the 2.5 and 97.5 percentile values equal to 103 ppm and 135 ppm, respectively.

To calculate 95% confidence intervals for the three U-Th dated and layer-count-adjusted ages at 63.5 mm, 47.2 mm, and 30.6 mm, we simulated 10,000 iterations of age calculations at each $^{230}Th/^{232}Th$ ratio value between 95 and 156 ppm. We began by randomly selecting an age drawn from the U-Th normal age distribution at 63.5 mm. We then checked if both the 47.2 mm age (77 years younger) falls within the U-Th normal age distribution at 47.2 mm and the 30.6 mm age (chosen from random selection from 151-156 years younger uniform distribution) falls within the U-Th normal age distribution at 30.6 mm. Any age solutions to these constraints were saved, and the mean and 95% CI were calculated from the solutions. The samples, their distance from the stalagmite top, their U-Th age calculated with an initial $^{230}Th/^{232}Th$ ratio of 117 ppm, and their layer-count-adjusted ages and 95% CI are provided in Supplementary Table 1. A complete table of the measured U-Th ratios and errors used to calculate the U-Th normal age distribution is provided in Supplementary Data 1.

Supplementary Table 1 | Stalagmite M1 spot ages. U-Th mean age is calculated using initial $^{230}Th/^{232}Th = 117 \pm 10$ ppm. The U-Th 95% confidence interval is calculated as the range of U-Th ages corrected using initial $^{230}Th/^{232}Th = 5 \pm 3$ ppm (lower bound) to initial $^{230}Th/^{232}Th = 170 \pm 10$ ppm (upper bound). Layer-adjusted mean age and 95% confidence intervals were calculated using the method described in the supplemental text. All ages reported in CE.

U-Th sample ID	Distance from stalagmite top (mm)	U-Th mean age (CE)	U-Th 95% CI lower bound	U-Th 95% CI upper bound	Layer-Adj mean age (CE)	Layer Adj. 95% CI lower bound	Layer Adj. 95% CI upper bound
MP1-U3	1.3	1900	1850	1920			
M1-sep1	11.2	1650	1560	1690			
MP1-U2	24.2	1610	1570	1630			
M1CA3	25.2	1610	1530	1650			
MP1-U4	30.6	1410	1320	1460	1400	1385	1415
MP1-U5	47.2	1310	1190	1370	1322	1307	1338
M1-sep7	63.5	1240	1210	1250	1247	1232	1263

M1 grew from ca. 1020 CE to 1410 CE in the lower calcite section below 28.0 mm from the top, and from ca. 1600 to 1930 CE in the upper aragonite section, from 26.6 mm to the top. The start and end dates were calculated using the COPRA method (Supplementary Figure 6[7]). The vertical extension rate of the lower calcite section is on average 0.2 mm/yr. The vertical extension rate of the aragonite section begins around 0.2-0.3 mm/yr, then gradually decreases to 0.03 mm/yr. We note the aragonite section age model is less constrained than the calcite section age model, given the lack of annual layers in the aragonite section (Supplementary Figure 6).

The hiatus between 28.0 and 26.6 mm spans approximately a 200-year period, from 1410 to 1600 CE. The two detrital layers at 28.0 mm and 26.6 mm (Supplementary Figure 4) might indicate two hiatuses (hiatuses 1 and 2) between the last age of the lower calcite section, and the first age of the upper aragonite section (1410-1600 CE). However, such sub-hiatuses currently cannot be resolved because of the uncertainties associated with the radiometric ages attempted within the dark calcite layer between 28.0 mm and 26.6 mm, and we indicate only a single hiatus at 26.6 mm. Additional U-series dates would be required to better constrain the depositional history of this interval.

The primary control on aragonite formation is drip rate[15]. Slower drip rates lead to increased supersaturation and prolonged degassing, which favors non-equilibrium formation of aragonite. The change from calcite to aragonite after the hiatus indicates either a change in local hydrology or climate (i.e., more arid conditions) that resulted in slower drip rates.

Stable isotopes ($\delta^{13}C$ and $\delta^{18}O$) were sampled at 0.5-mm intervals and measured using a Thermo Fisher Scientific Gasbench coupled to a Delta V isotope ratio mass spectrometer in the Godwin Laboratory, Cambridge University (Supplementary Figure 6). Values in the top section were corrected for the difference in fractionation factors between aragonite-water and calcite-water. Oxygen isotope ratios ($\delta^{18}O$) were corrected by -0.88‰ relative to calcite[16]. We further corrected the $\delta^{18}O$ in the aragonite section by +0.377‰ to account for the difference in the acid fractionation factor between calcite and aragonite at 70°C. Carbon isotope ratios ($\delta^{13}C$) were adjusted by -1.2‰[16].

Comparison of the $\delta^{18}O$ record of M1 to the Chaac $\delta^{18}O$ record from Tzabnah Cave[4], located 11.2 km north of Mayapan, shows that the $\delta^{18}O$ record of M1 displays higher and less variable values. Because M1 was located near the cave entrance, evaporation probably accounts for the greater $\delta^{18}O$ values. The $\delta^{18}O$ record shows a sharp increase just prior to the hiatus (values between -5‰ and -3‰), and thereafter the mineralogy transitions from calcite to aragonite (Supplementary Figure 6).

Similarly, highest $\delta^{13}C$ values occur just prior to the hiatus and within the top section (-5 – -6‰) of the speleothem (Supplementary Figure 6), which may indicate increased kinetic fractionation, associated with prior carbonate precipitation in the epikarst and/or cave, and reduced vegetation and soil microbial activity above the cave during this later interval[17,18]. All these processes are connected and indicative of drier conditions.

Dirt layers in the lower section are most prominent at the rim of the stalagmite, whereas the apex is fairly clean (Supplementary Figure 6). This feature is explained by flood waters that filled the cave during particularly wet times and covered the stalagmite with thin clay layers, which were washed from the apex when the water receded and normal dripping resumed. The cave geometry is such that surface runoff can enter the opening of Cenote Ch’en Mul and travel downslope through the cave until it reaches the water table at the far end. Water may pond locally in the central chamber during flood events. The lower section of the speleothem seems to have been deposited during times of

frequent flooding, whereas the upper aragonitic section is clean and generally lacks these flood layers (with a few exceptions). It is likely that human activity near and directly above the cave influenced local hydrology, which might explain the substantial input of detrital material in the lower segment of stalagmite M1.

Local paleolimnological evidence

Supporting evidence for drying around 1450 CE was also found in lake sediments from Aguada X'caamal, located only 27 km west of Mayapan[19]. The approximately 4-m-long sediment core spans the last ca. 3500 years (Supplementary Figure 7). We updated the ^{14}C-based age model using the INTCAL20 calibration curve[20] and OxCal v.4.4.2[21] Poisson process deposition model[22], with a variable k parameter (rigidity), set as k_0=1 cm^{-1} with variability allowed between a factor of 10^0 and 10^2 in[23].

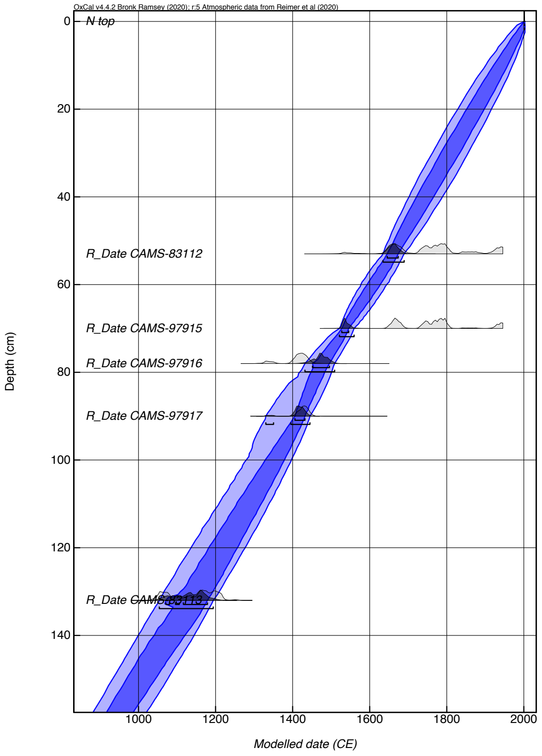

Supplementary Figure 7 | Radiocarbon-based age model of the Aguada X'caamal sediment core. Updated using IntCal20 and Oxcal v. 4.4.2.

Ostracod (*D. stevensoni*) and gastropod (*P. coronatus*) δ^{18}O values increase abruptly by >2 ‰ at 88 cm below lake floor (Supplementary Figure 7), suggesting a marked change in lake hydrology. This increase was accompanied by a faunal change, marked by the first appearance of the benthic foraminifer *Ammonia beccarii parkinsoniana,* which is usually a marine species, but tolerates a wide range of salinities (7-67 ppt) and is common in coastal lakes with high ion concentrations. At the same sediment depth (88 cm), charophyte remains that were abundant below this level vanish because the macroalgae are intolerant of high salinity (Supplementary Figure 8).

The updated age model for Aguada X'caamal suggests the sharp changes at 88 cm occurred at ~1420 CE (with a 1σ confidence interval (CI) of 1413–1441 CE and 2σ CI of 1345–1458 CE). The isotopic and faunal changes in Aguada X'caamal occurred at about the same time as the cessation of growth in stalagmite M1 from Mayapan (1410 ±17 2σ CE). The transition from calcite to aragonite deposition above the hiatus in stalagmite M1 (Supplementary Figure 5) is consistent with the increase in mean $\delta^{18}O$ values and persistent occurrence of the benthic foraminfer *A. beccarii* in the upper part of the sediment profile of Aguada X'caamal (Supplementary Figure 7).

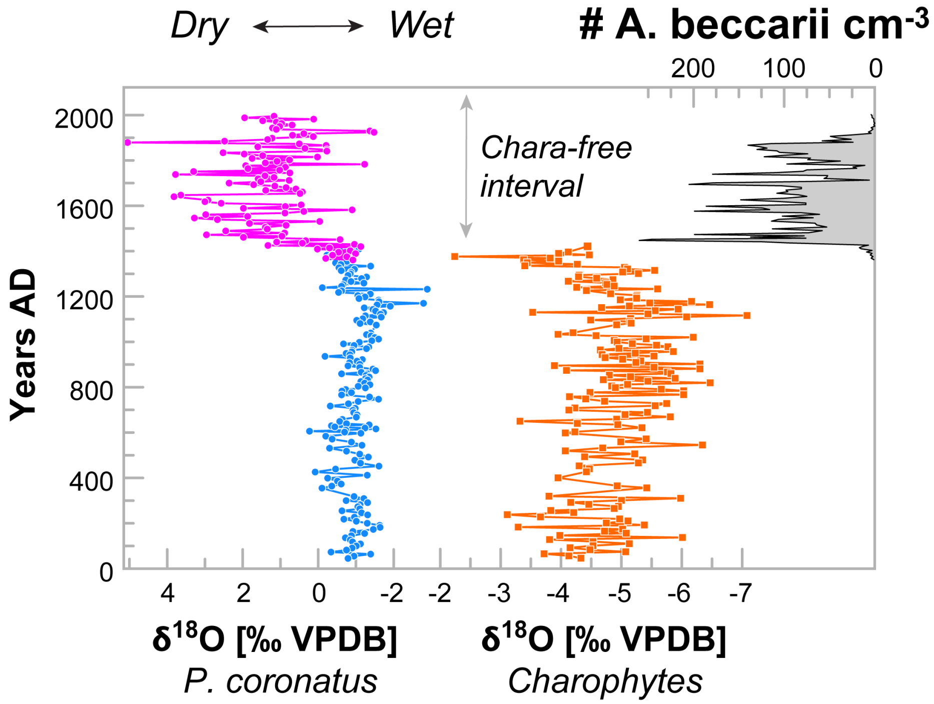

Supplementary Figure 8 | Evidence of changes in water chemistry and related ecological changes in Aguada X'caamal, near Mayapan. Pink-colored P. coronatus data come from sediment core MWI1, whereas blue-colored data come from overlapping core MWI. A. beccarii (grey) appears at the same time that charophytes (orange) disappear in the record. Data from[19]. All data provided in Source Data File.

Supplementary Note 3:
BURIAL CONTEXTS

DISTINGUISHING VIOLENT DEATHS FROM PROPER BURIALS

Many proper respectful burials have been excavated at Mayapan. This enabled us to distinguish anomalous burials that clearly deviate from established reverential funerary treatment (see[24–30]). Burials have been excavated at Mayapan by the Carnegie Institution of Washington (CIW), the Instituto Nacional de Antropología e Historia of Mexico (INAH; associated with the Proyecto Mayapan, the Salvamento Arqueológico en la Modernización de la Carretera Mérida-Mayapan-Oxkutzcab and the Salvamento Arqueológico Modernización de la Carretera Mérida-Chetumal), the Proyecto Económico de Mayapan (PEMY), the Mayapan Taboo Cenote Project and as part of the dissertation research of Brown[31].

We identified violent death employing a combination of osteological and archaeological criteria, including perimortem cranial fractures and sharp force trauma, embedded points, cutmarks indicative of defleshing or dismemberment, and anomalous burial patterns. Within this grouping, we further distinguished between cases of violent death caused by civil conflict and those caused by state-sanctioned violence carried out by Mayapan’s warriors and priests. Violent death from civil conflict was identified on the basis of mass burials found in association with evidence of deliberate destruction, or “desecratory termination rituals”[32,33] (also see[24]). Three mass burials have been identified at Mayapan to date. These are designated Mass Burials 1, 2 and 3. Whereas at other Maya sites such evidence could reflect invasion by a foreign force, Mayapan’s dominance in terms of size[34] and military strength makes this scenario unlikely. Mayapan's population was an order of magnitude larger than its contemporaries, and no other large Postclassic sites are located in its vicinity. It was a militaristic state from the time it was founded in the latter 12th century. Violent death attributable to state-sanctioned violence. Violent death attributable to state-sanctioned violence was identified on the basis of war captives from Mayapan's military campaigns, individuals who were periodically brought back to the city for sacrifice by the city's priests and placed as offerings in dedication to the city's patron deities, as identified in the skull niche in the façade of Q.162a, the two alleys adjacent to principal round temple Q.152, and sacrificial burial shaft temples Q.95 and Q.58[35–40]. Each of these contexts is described in detail below; for a listing of osteological determinations and direct radiocarbon dates see Supplementary Data 2. Subsequent sections provide an overview of the many proper respectful burials excavated at Mayapan.

BURIAL CONTEXTS: VIOLENT DEATHS

Mass Burial 1

Mass burial 1 (MB1; Supplementary Figure 9) was partially excavated by the Carnegie Institution of Washington (CIW) in 1953 (Supplementary Figure 10a) and by Instituto Nacional de Antropología e Historia (INAH) archaeologists in 1996 at the principal (northwestern) entrance to the site's main plaza[40,41] (Supplementary Figure 10b-d). MB1 was encountered within 30 cm of the surface in a zone of ash, and covers an extensive area west and north of shrines Q.79 and Q.79a. It consists of eight articulated skeletons and numerous isolated bones. Portions of smashed Chen Mul effigy incense burners, the most prominent type of ritual pottery at the site, were mixed in with the remains. The haphazard arrangement of the articulated skeletons suggested violent death of these individuals to the excavators[40,41]. Flint knives were found in the thoracic cavities of two of these skeletons and in the pelvic cavity of a third[41] (Supplementary Figure 10a). A fourth skeleton (Burial 21) has a flint arrowhead tip embedded in the right scapula (Supplementary Figures 10b and 10c; also see[27]). An isolated left fibula (Burial 18) exhibits cut marks resulting from the dismemberment and sectioning of the bone for the purpose of trophy-taking (Supplementary Figure 10d). Individuals interred in MB1 may represent victims of internal strife[42]. Carnegie archaeologists originally proposed that it relates to the violent overthrow at the end of Mayapan's occupation[41], but an earlier round of radiocarbon analyses[43] demonstrated that it predates the site's collapse.

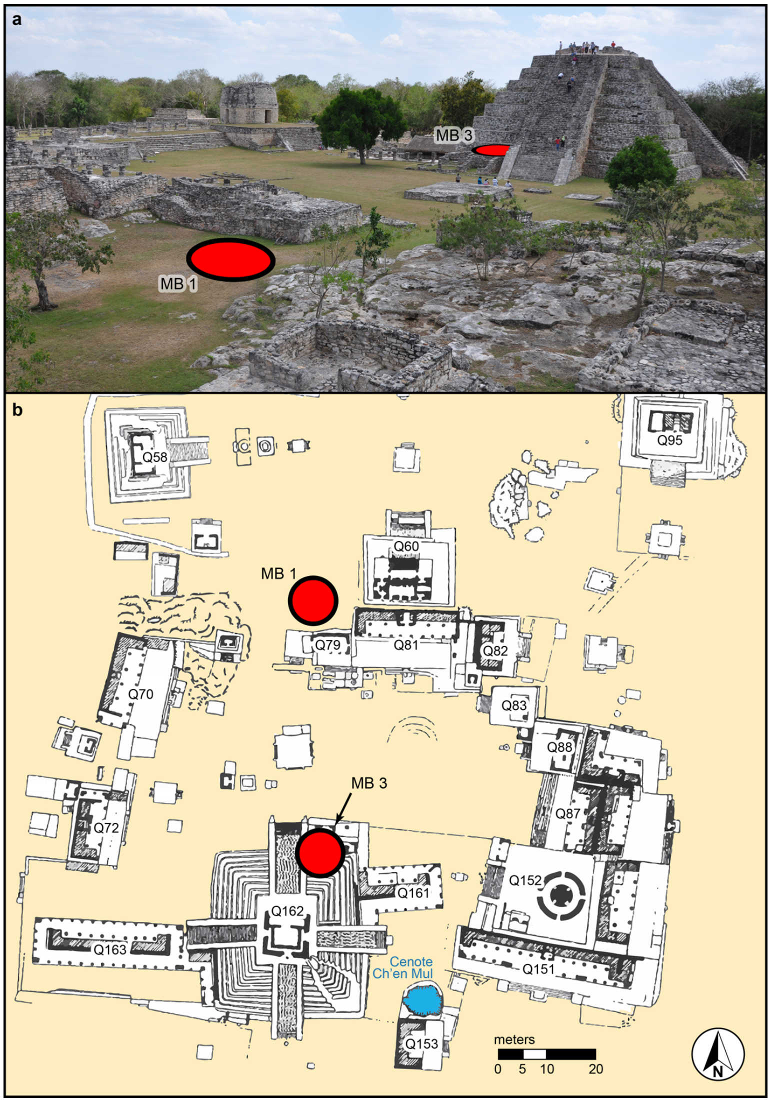

Supplementary Figure 9 | Locations of mass burials MB1 and MB3. a Photograph showing the location of MB1 and MB3 in the center of Mayapan (red) **b** Planview map of the central precinct of Mayapan showing locations of MB1 and MB3 in red and the dry Cenote Ch'en Mul in Blue.

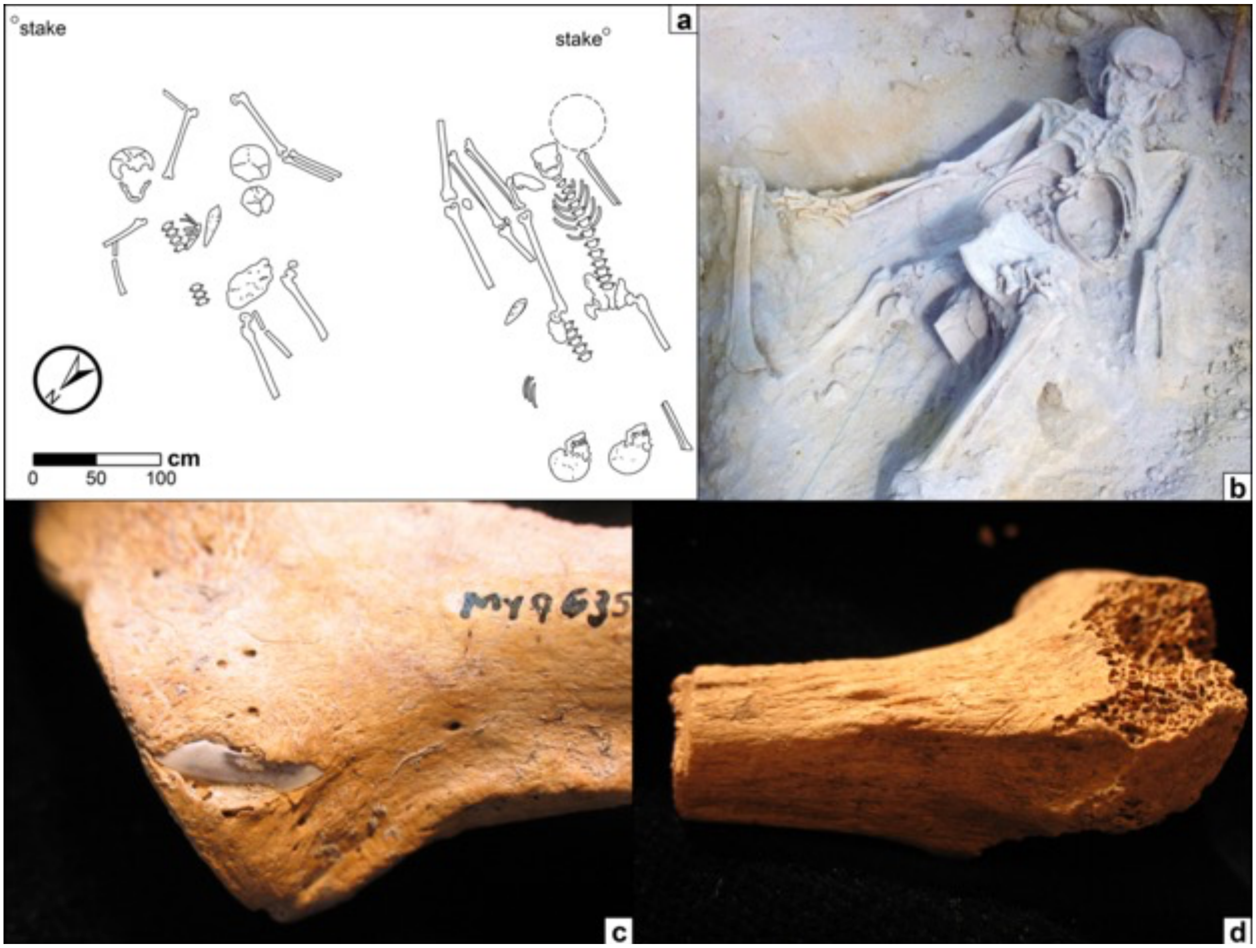

Supplementary Figure 10 | Mass Burial 1. a Plan drawing illustrating the position of articulated skeletons in the portion of Mass Burial 1 excavated by the Carnegie Institution of Washington, modified from [41]; **b** Burial 21 *in situ* photograph; **c** Burial 21 embedded tip of flint arrowhead in right scapula; **d** Burial 18 postero-lateral view of isolated proximal left fibula exhibiting cut marks resulting from dismemberment and sectioning of the bone for the purpose of trophy-taking. *In situ* photograph by P.D.K. and B.E.O. Photographs of traumatic lesions by S.S.

Mass Burial 2

Mass burial 2 (MB2) was excavated by archaeologists from the University at Albany-SUNY and INAH in 2003 and 2008 (PEMY) at the Itzmal Ch'en group[24]. This group is located in Mayapan's northeastern corner and is the second largest concentration of ritual and administrative buildings at the site. The mass burial was encountered 10 cm below the surface, spread out over an extensive area near the southwest edge of the group's platform and its principal staircase[24]. MB2 consists primarily of burned and butchered fragments of human bone intermingled with sherds from smashed Chen Mul effigy incense burners. Eighty-five percent of the associated lithic tools are projectile points, an unusually high concentration of weaponry that suggests this mass burial was produced by an act of war[24,42]. A minimum number of individuals (MNI) of 20 was

calculated from dental remains. The only indications of subadults are a single deciduous tooth and two incompletely developed permanent teeth. The Itzmal Ch'en group's temple (H.17) and colonnaded hall (H.15) exhibit evidence of deliberate destruction and ritual termination, the latter through placement of portions of smashed Chen Mul effigy incense burners on the floors of these structures. This may have occurred around the same time that MB2 was deposited. Taken together, the evidence suggests that the human remains are those of the Itzmal Ch'en group's elite patrons[24,42].

Mass Burial 3

Mass Burial 3 (MB3) was excavated by INAH archaeologists in 1996 at temple Q.162, or El Castillo de Kukulkan, Mayapan's principal pyramid[40]. MB3 was encountered directly east of the main (north) staircase of Q.162, in the passageway that separates it from small structure Q.162c (Supplementary Figures 11a-g). This mass burial consists of the skeletal remains of at least nine individuals, represented mainly by crania as well as isolated limbs, mixed with building collapse that filled the passageway to a height of approximately 125 cm above the highest floor[40,44]. This includes seven subadults between two and fifteen years-of-age at death and two young adult males between 18 and 35 years-of-age at death. Most remains were disturbed; only the young adult male at the base of the deposit (Burial 11) was found fully articulated and largely complete (Supplementary Figures 11d and 11e). This individual exhibited a dorsal extended position and was located approximately 30 cm above the highest floor. The nature of the remains in MB3 and their disposition, coupled with the lack of associated grave goods, led the excavators to suspect violent death[40]. This was confirmed through osteological analysis which identified perimortem rib puncture wounds from stabbing or being shot with a pointed weapon, such as a knife, spear, dart or arrow, in the complete young adult male (Burial 11), as well as the subadult (Burial 10) found directly above this individual [44] (Supplementary Figures11f and 11g). The presence of building collapse and the lack of a floor above in the most sacred context at the site, point to the placement of these remains around the time of Mayapan's abandonment[40,45]. This has now been confirmed through direct dates on human bone from these nine skeletons. Primary Burial 11, around which MB3 appears to have been centered, produced a date of 430 ± 20 BP (UCIAMS-142304), or 1430–1480 cal. CE (2σ). A very similar date was obtained for nearby Burial 25 (Lab # UCIAMS-135070, 405 ± 15 BP, 1440–1610 cal. CE [2σ]), an old female whose body was haphazardly strewn directly west of the main staircase of Q.162 (Supplementary Figure 11h). Whereas MB3 was found well above any floors, Burial 25 was directly over a plaster floor, indicating the Mayapan polity had not yet, or had only recently, collapsed at the time of its placement. The late date of MB3, its placement beside the site's principal public building and mitochondrial DNA evidence suggesting that these individuals were potentially related on the maternal line are consistent with historical accounts of the massacre of members of the ruling Cocom family[46]. Further,MB3 closely parallels a mass burial in a late context at contemporary Champotón (Pit 2, Group X[47]). The latter was also deposited beside an important structure and contained adult males and subadults of similar ages who show evidence of violent death, suggesting that the events surrounding the collapse of these two important Late Postclassic sites were linked.

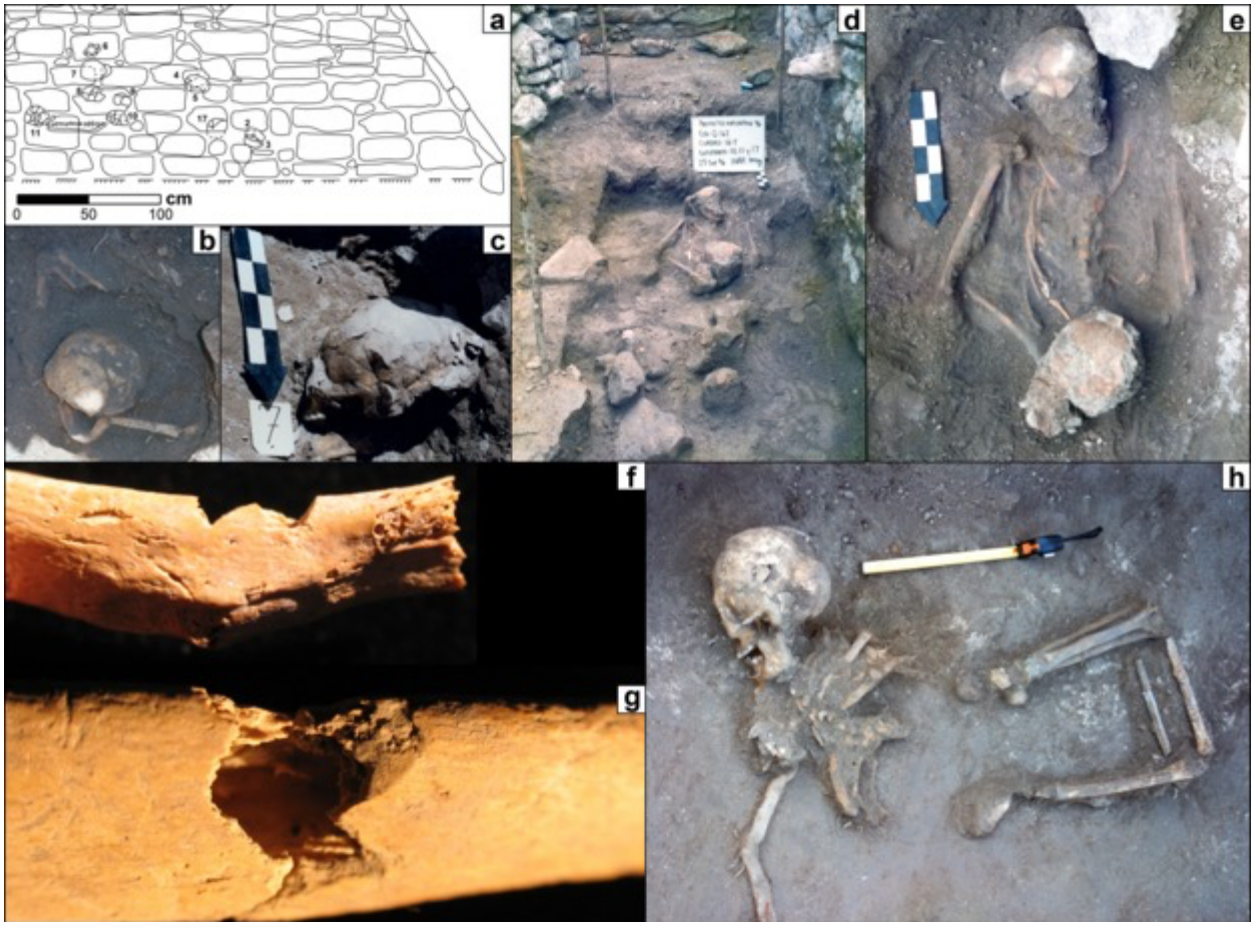

Supplementary Figure 11 | Mass Burial 3. a Profile drawing illustrating the position of articulated and disarticulated skeletons; **b** Burial 2 *in situ* photograph; **c** Burial 7 *in situ* photograph; **d** Burial 10, 11 and 17 *in situ* photograph; **e** Burial 10 and 11 close-up *in situ* photograph; **f** Perforating traumatic lesion on Burial 10 left fourth rib; **g** Perforating traumatic lesion on Burial 11 right third rib; **h** Burial 25 *in situ* photograph. *In situ* photographs and drawings by M.G.E, C.G.A., B.E.O., P.D.K. Photographs of traumatic lesions by S.S.

Q.162a Skull Inside Niche

Cranial remains of an old adult of probable male sex were found in the head niche of a death god or skeletal warrior stucco sculpture in the façade of Q.162a, the substructure of El Castillo de Kukulkan, the preeminent religious structure at the site (Supplementary Figure 12)[35,37,40] (Supplementary Figure 12). Traces of stucco remain on the mandible. These cranial remains were directly dated (PSUAMS-1169) to 910 ± 20 BP, or 1040–1215 cal. CE (2σ). Peraza et al.[43] report a similar date for this structure (A-12793) of 930 ± 30 BP, or 1030–1205 cal. CE (2σ), based on dating of charcoal from fill that comes from one construction phase earlier, but the direct date on human bone confirms that by the latter 12th century, Mayapan was already an important political center. This is

also the earliest directly dated human bone from any civic-ceremonial structure at the site.

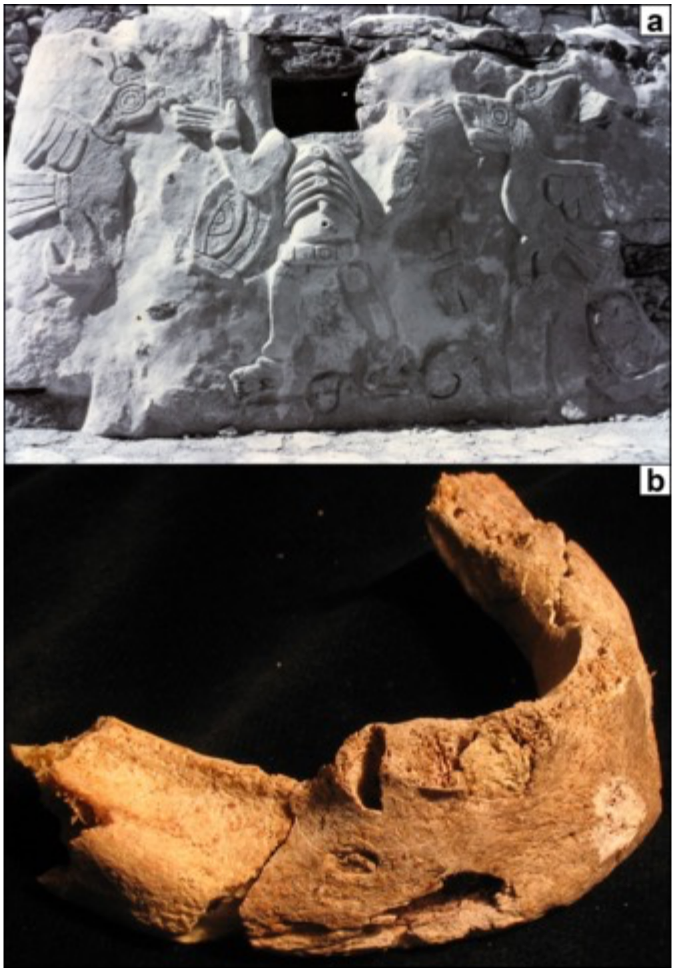

Supplementary Figure 12 | Q.162 Skull Inside Niche. a Q.162a stucco façade niche that contained cranial remains; **b** Mandible from the niche, exhibiting traces of stucco in the mental foramen and RC 1 alveolar bone. Q.162a stucco façade photograph by P.D.K. Photograph of mandible by S.S.

Q.152/Q.152c Sacrificial Alley

This deposit was excavated by INAH archaeologists in 1997 at structure Q.152, Mayapan's main round temple[37] (Supplementary Figure 13). It was encountered in the passageway east of Q.152 that separated it from colonnaded hall Q.152c. Burial 29, which forms the bulk of the deposit, was placed directly on the plaster floor of the passageway in alignment with the eastern doorway of the round temple (Supplementary Figure 13a). The passageway was sealed following its placement[37]. Additional human remains were recovered elsewhere in the passageway. This deposit consists of the secondary remains of at least 20 individuals represented mainly by crania (n=20) and long bones (right femurs are the most common, n=8[25]). Age-at-death is 16 years or older for all but one individual. Sex could be determined in 13 crania: 9 are males whereas 4 are females. Skeletal trauma is common in this deposit and includes three cases each of antemortem and perimortem cranial trauma[27] (Supplementary Figure

13b). Additional human remains recovered elsewhere in the passageway also exhibit healed and unhealed cranial trauma. Furthermore, the presence and distribution of cut marks indicates that at least some individuals were defleshed and dismembered[25] (Supplementary Figures 13c and 13d). Associated artifacts include a stingray spine, bone awl, obsidian prismatic blades and numerous pottery sherds, though none from Chen Mul effigy incense burners. The individuals interred in this deposit were likely captured during military campaigns waged by Mayapan's warriors and brought back to the site for sacrifice by the city's priests.

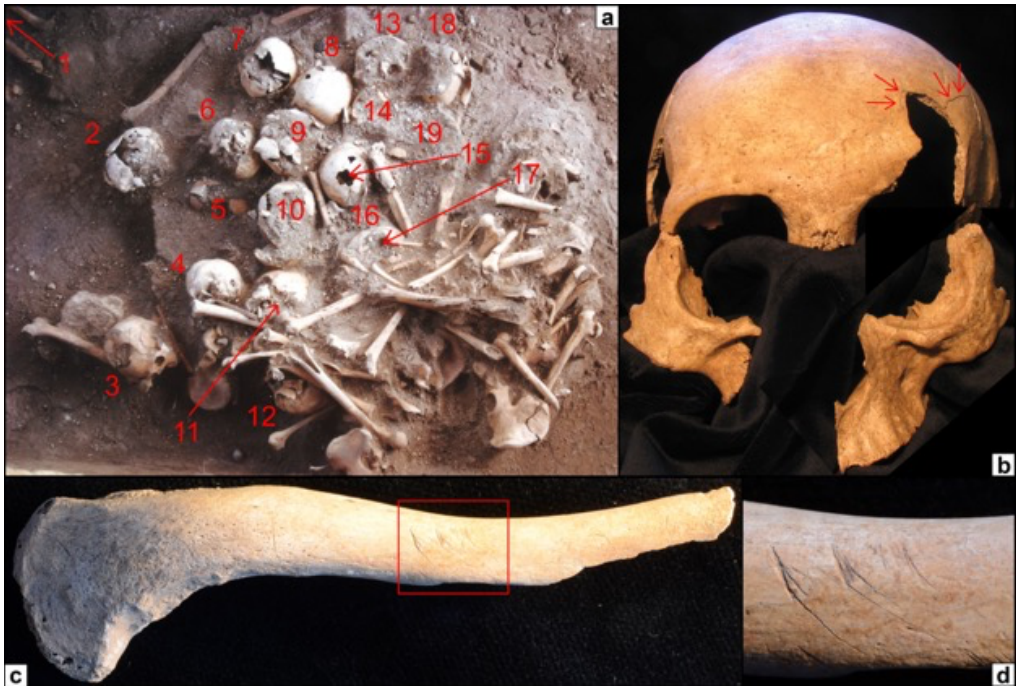

Supplementary Figure 13 | Q.152/Q.152c Sacrificial Alley. a Burial 29 *in situ* photograph with crania labelled; **b** Burial 29 Cranium 1 frontal view illustrating perimortem depressed fracture with concentric and radiating fracture lines of left supraorbital region of the frontal bone; **c** Superior view of right clavicle exhibiting cutmarks caused by defleshing; **d** Close-up of cutmarks in right clavicle shown in c. *In situ* photograph by P.D.K. Photographs of traumatic lesions by S.S.

Q.152/Q.151 Sacrificial Alley

This deposit was partially excavated by Carnegie Institution of Washington archaeologists in the 1950s and later by INAH archaeologists in 1997 in the passageway separating Q.152 and colonnaded hall Q.151, the Hall of the Chac Masks, to its south[37,39]. This deposit consists of the secondary remains of at least 5 adults represented mainly by crania and long bones. Two crania were recovered with the first one or two cervical vertebrae still in articulation. Associated artifacts include a stingray spine, shell, faunal remains, stalactites, stucco, two *manos*, lithics and numerous pottery sherds. This deposit closely resembles that found in the Q.152/Q.152c Sacrificial Alley and likely represents a similar ritual activity, specifically the sacrifice of

war captives. Human bone excavated by INAH archaeologists was directly dated (UCIAMS-181696) to 685 ± 20 BP, or 1275–1385 cal. CE (2σ). Both of the sacrificial alley deposits associated with Q.152 date to the period of peak population and construction activity that preceded the drought that began in the middle of the 14th century, and underscore the importance of military activity in the development and maintenance of the Mayapan State.

Q.95 Sacrificial Burial Shaft

Sacrificial burial shafts are unique to Mayapan, and have been excavated in four temples or shrines. The shaft in Q.95, The Temple of the Fisherman, was present from the first of the temple's four major construction phases[38]. The shaft is bottle-shaped in cross-section and approximately four meters deep. The shaft was completely excavated by Carnegie Institution of Washington archaeologists in 1953 and contained at least 41 skeletons that represent individuals of all ages and both sexes and are intermingled with faunal remains, pottery sherds, shell, metal and charcoal[38]. Near the top of the shaft was a tapered stone altar similar to those upon which splayed victims were sacrificed, as depicted in numerous scenes in the Postclassic codices and painted ceramic vessels of the Classic period. The gradual accumulation of these victims and long-term use of the shaft is indicated by stratigraphic evidence combined with the absence of Chen Mul effigy incense burner sherds in the lowest level, but high numbers of such sherds in the upper levels. Beneath and in direct alignment with the shaft is a small subterranean chamber that contained the skeletons of an adult male and female. These skeletons were found on the floor of the chamber and were covered with large quantities of faunal remains within which were mixed pottery sherds, artifacts of bone, shell, and stone, and the disarticulated remains of two children. Placed on top were two complete ceramic vessels. This was followed by the sealing of the chamber with a capstone and construction of the shaft and temple directly above it. Human bone from the male on the subterranean chamber floor was directly dated (PSUAMS-1139) to 820±20 BP, or 1175–1270 cal. CE (2σ), providing a *terminus post quem* for the construction of Q.95, which postdates the construction of Q.162a. Human bone from the shaft above the chamber was directly dated (PSUAMS-1136) to 685±20 BP, or 1275–1395 cal. CE (2σ).

Q.58 Sacrificial Burial Shaft

The sacrificial burial shaft in temple Q.58 (The Crematorium) is bottle-shaped in cross-section and was present from the first construction phase of the structure, as with Q.95, though is deeper at approximately six meters[38]. Most of the contents of the shaft had been disturbed by looting, whereas the remainder, excavated by Carnegie Institution of Washington archaeologists in 1953, consisted principally of the partially burned bones of at least four subadults and adults, and faunal remains. Extensive excavations by INAH archaeologists in 2001 uncovered many additional fragments of human bone strewn about the surface of the temple, which may have originally been inside the shaft[48,49]. Two of these bone fragments were directly dated (UCIAMS-161688 and PSUAMS-1258) to 830±20 BP (1175-1270 cal. CE [2σ]) and 675±30 BP (1275-1395

cal. CE [2σ]), respectively. These date ranges suggest that Q.58 is of comparable antiquity to Q.95.

BURIAL CONTEXTS: COMMONER BURIALS

Commoners Inside Center: Q.95 Small Cemetery

A concentration of seven burials (Burials 40–46), representing seven individuals of all ages and both sexes, were interred directly in the soil behind small residential structures Q.92 and Q.93, and excavated by INAH archaeologists in 2001[50]. The location of these humble structures near Q.95, the Temple of the Fisherman, suggests they were occupied by custodians or servants to the elite patrons of this temple. Direct dates on human bone from five of these seven burials, however, indicate that utilization of this small cemetery was initiated prior to the construction of the temple and continued throughout the main period of site occupation. Two of the older dates (UCIAMS-142321, 920±20 BP, 1035–1200 cal. CE [2σ]; UCIAMS-135074, 830±15 BP, 1175–1265 cal. CE [2σ]) pertain to disarticulated burials (Burials 43 and 40, respectively), whereas the two more recent dates (UCIAMS-135075, 675±15 BP, 1280–1385 cal. CE [2σ]; UCIAMS-142320, 650±20 BP, 1285–1395 cal CE [2σ]) correspond to articulated burials (Burials 46 and 41, respectively). This is to be expected, for sequential interment of later burials resulted in disturbance of older ones, though an additional older burial (Burial 45, UCIAMS-161693, 910±20 BP, 1040-1215 cal. CE [2σ]) was still in articulation when encountered. Two *tecomate* miniature vessels (one Navula Unslipped, 1 Mama Red), containing copper metallurgical production debris[51] and a cut fragment of cranial vault, were found nearest to Burials 40, 41 and 43. The vessels may have constituted grave goods, although it is unclear with which of these three non-contemporaneous burials they were associated. Burials 34 and 36 were excavated within small residential structure Q.94, which may have served a function similar to that of Q.92 and Q.93.

Commoners Inside Center: Q.58 Small Cemetery

Five burials (Burials 50-54), representing six individuals of all ages and both sexes, interred directly in the soil near small residential structures Q.67 and Q.68, were excavated by INAH archaeologists in 2002[50]. As with the Q.95 small cemetery, the location of these humble structures near an important temple, in this case Q.58 or The Crematorium, suggests they were occupied by custodians or servants to the elite patrons of the temple. Direct dates on human bone from all six skeletons span much of the main period of occupation of the site. As with the Q.95 small cemetery, the oldest sample (UCIAMS-161692, 970±20 BP, 1020–1155 cal. CE [2σ]) dates to the initial establishment of an important political center at the site and predates the nearby temple, further reinforcing the antiquity of this burial practice. Nearby burials (Burials 47–49) were also single, flexed interments, which although found closer to Q.58, appear to reflect a similar mortuary function.

Commoners North of Center

Five burials, representing 14 individuals of all ages and both sexes, were excavated under or near commoner residences in Quadrant L, north of the site's monumental center. Two burials were excavated by the Proyecto Económico de Mayapan Project (PEMY) and INAH archaeologists in 2003 (Burials 03-03 and 03-05)[52], whereas the remainder were excavated by INAH archaeologists in 2015 (Burials 1, 2 and 3). Direct dates on human bone from 12 of these skeletons span the entire range from the Terminal Classic (Burial 03-03, UCIAMS-136062, 1235±25 BP, 680–885 cal. CE [2σ]) until the final period of occupation of the site (Burial 1, PSUAMS-1172, 560±20 BP, 1320–1425 cal. CE [2σ]).

Commoners East of Center

Seventeen burials, representing 35 individuals of all ages and both sexes, were excavated under or near commoner houses in the large, dense residential area east of the site's monumental center, mainly found in Quadrants R and Y, though also in parts of Quadrants K and X. One burial was excavated by Carnegie Institution of Washington archaeologists in the 1950s (Burial 10; Smith 1962), and INAH archaeologists excavated four burials in 1997 (Burials 1, 2, 11 and 12)[53] and 10 burials in 2015 (Burials 4, 5, 6, 7, 8, 9, 10, 12, 21 and 26). PEMY and INAH archaeologists excavated two burials in 2003 (03-06 and 03-07)[52]. Direct dates on human bone from 18 of these skeletons span from Mayapan's initial founding as an important political center (Burial 1, UCIAMS-142127, 875±20 BP, 1050–1225 cal. CE [2σ]) until the final decades of the site's occupation in the 15th century (Burial 10, PSUAMS-1137, 490±15 BP, 1410–1445 cal. CE [2σ]).

Commoners Near Itzmal Chen Group

Five burials, representing six adults of both sexes, were excavated near commoner houses in Quadrants H, I and F in Mayapan's northeastern periphery, near the Itzmal Chen group, the second largest concentration of public architecture at the site. PEMY and INAH archaeologists excavated one burial in 2003 (Burial 03-04), two in 2008 (Burials 08-01 and 08-02) and two in 2009 (Burials 09-01 and 09-02)[52,54]. Direct dates on human bone from four of these skeletons all predate the 14th century, with the oldest dating to the Late Classic (Burial 03-04, UCIAMS-140857, 1270±20 BP, 670–800 cal. CE [2σ]). The next oldest burial (08-02 Individual 1, PSUAMS-1180, 925 ± 20 BP, 1035–1175 cal. CE [2σ]) was interred with a portion of a Provincia plano-relief (Fine Orange) vessel, a Late/Terminal Classic type[55], produced in the Jonuta-Tecolpan region of eastern Tabasco during the period AD 750-900[56,57]; it was likely an imported commodity and also an heirloom, interred at least a century after its likely manufacture. The dates of the interments suggest that the Terminal Classic in the Northern Maya Lowlands extended into the 11th century, which has long been suspected, but the present study represents the first time it has been reliably demonstrated through radiocarbon dating. This old adult male exhibits a healed weapon cranial wound in the left supraorbital region of the frontal bone[27].

BURIAL CONTEXTS: ELITE BURIALS

Elites Inside Center: Shrines

An ossuary containing the cranial remains of at least nine individuals of all ages and both sexes, was excavated below the floor of shrine Q.88c in 1997 by INAH archaeologists[40]. Aside from crania, the only remains present were four mandibular teeth and fragments of a left humerus and right pelvis[25]. Grave goods included a Mama Red tripod dish and a *mano* from a *metate*. This shrine is prominently located in Mayapan's main plaza and has a doorway facing temple Q.162, which would have permitted reentry. Direct dates on human bone from seven of these individuals are evenly spread across the main period of occupation of the site in the 13th and 14th centuries. The extended period of time over which remains were deposited in this shrine supports the interpretation that this represents an elite mortuary practice associated with ancestor veneration[25,42]. Collectively, direct dates on human bone from five individuals interred in ossuaries in shrines Q.89, Q.90, Q.140 and Q.149 were roughly contemporaneous.

Elites Inside Center: Halls

Ten burials (Burials 28, 30, 31, 32, 55, 56, 57, 58, 62, and 63), representing 16 individuals of all ages and both sexes interred below colonnaded halls Q.152c, Q.72, Q.54 and Q.99 or, more typically, below adjacent plaza floors, were excavated by INAH archaeologists[40,50,53,58,59]. The function of these enigmatic structures has been debated since the 1950s, though analysis of the extensive data resulting from large-scale excavations by INAH archaeologists since 1996 indicates they were used for elite ritual and political purposes, and perhaps even "were seats of political power that embodied the factions who built and used them," as is documented ethnohistorically for similar structures at the Postclassic K'iche' capital of K'umarcaj (Utatlán) in highland Guatemala[42]. Direct dates on human bone from 10 skeletons span from the initial establishment of an important political center at the site (Burial 28, UCIAMS-142125, 955±20 BP, 1030–1160 cal. CE [2σ]) until the final decades of the site's occupation in the 15th century (Burial 55, PSUAMS-1125, 520±20 BP, 1400-1440 cal. CE [2σ]), though most date to the late 13th–14th centuries.

Elites West of Center

Ten burials, representing 14 individuals of all ages and both sexes, were excavated under or near elite residences in the crafts barrio immediately west of the site's monumental center by PEMY and INAH archaeologists in 2003 (Burials 03-01, 03-02 and 03-09) and 2009 (Burials 09-03, 09-04, 09-05, 09-06, 09-07, 09-08 and 09-09)[52,54]. Direct dates on human bone from eight of these skeletons are evenly spread across the main period of occupation of the site in the 13th and 14th centuries. The oldest date (Burial 09-06, UCIAMS-135079, 795±15 BP, 1220–1270 cal. CE [2σ]) pertains to an infant approximately 15 months of age-at-death, who was interred at the bottom of a deep midden adjacent to elaborate residence Q.176a. The most recent date (Burial 09-

04, UCIAMS-136065, 605±25 BP, 1300–1405 cal CE [2σ]) does not extend into the 15th century, suggesting that the depopulation of this barrio had already begun prior to the site's collapse.

Elites East of Center

Twenty-four burials, representing 39 individuals of all ages and both sexes, were excavated under or near elite houses in the large, dense residential area east of the site's monumental center in Quadrants R and Y. One burial (31) was excavated by Carnegie Institution of Washington archaeologists in the 1950s[28], whereas INAH archaeologists excavated 10 burials in 1997 (Burials 3, 4, 5, 6, 7, 8, 9, 10, 13 and 14[53]) and 13 burials in 2015 (Burials 11, 13, 14, 15, 16, 17, 18, 19, 20, 22, 23, 24 and 25). Direct dates on human bone from 22 of these skeletons span from before Mayapan's founding as an important political center (Burial 14, PSUAMS-1241, 1025±20 BP, 990–1035 cal. CE [2σ]) until the final decades of the site's occupation in the 15th century. Three out of four burials in R.183b, which contained a large cache of copper bells and metallurgical production debris, date to the second half of the 13th century, whereas R.183a, a shrine in the same residential compound, had two of the most recent burials (Burial 11 Individual 2, PSUAMS-1426, 510±25 BP, 1400–1445 cal. CE [2σ]; Burial 15, PSUAMS-1426, 465±35 BP, 1405–1480 cal. CE [2σ]).

BURIAL CONTEXTS: INDETERMINATE

Cenote San José

Cenote San José is located in Quadrant EE, approximately 500 m outside Mayapan's wall, directly south of the monumental center, and contains the submerged, disarticulated remains of many individuals. Cenotes are abundant at the site, but it is very unusual for them to contain human skeletons. In 1998 INAH archaeologists recovered the remains of at least 20 individuals, principally represented by crania, which was only a small subset[29]. All but one of these individuals is adult and both males and females are represented. The frequency of healed fractures in these crania is unusually high and comparable to that observed in the Sacred Cenote of Chichen Itza, though unlike the latter, there is no evidence of sacrifice. Excavations discovered the original stairs that lead down to the water, which were sealed in the Postclassic period, suggesting a pre-Hispanic date for most of the remains, though a hole in the cenote roof and the presence of several cow skulls left open the possibility that some had been introduced after European contact. Direct dates on human bone from 13 of these skeletons confirm that the use of this cenote as a mortuary space occurred mainly, perhaps solely, during the Postclassic period. The earliest date (CR6, PSUAMS-1084, 875±20 BP, 1050–1225 cal CE [2σ]) is roughly contemporaneous with the initial founding of an important political center at the site, but all other dates fall within the latter 13th and 14th centuries, indicating the short duration of this mortuary practice.

Cenote Sac Uayum

Cenote Sac Uayum is located in Quadrant X, a short distance outside and south of Mayapan's city wall. Underwater archaeological investigations conducted in 2013 as part of the Mayapan Taboo Cenote Project discovered substantial numbers of human skeletons, making it the only such cenote at the site, other than Cenote San José[60]. Direct dates on three human bones also confirm the contemporary use of these two cenotes as mortuary spaces.

Problematic Deposits

Small human bone scatters that do not contain enough skeletal material or associated artifacts to enable reconstruction of their function are common in and around public buildings in Mayapan's monumental center, particularly temples and colonnaded halls, and were not assigned burial numbers by the excavators. Direct dates on human bone from 19 such contexts span from Mayapan's initial founding as an important political center (PSUAMS-1256, 960±30 BP, 1025–1160 cal. CE [2σ]) to the final decades of the site's occupation in the 15th century and possibly beyond (PSUAMS-1159, 365±20 BP, 1455–1635 cal. CE [2σ]), although 15 out of 19 samples have dates that cluster in the late 13th and 14th centuries.

Supplementary Note 4:
STATISTICAL MODELS

SUMMED PROBABILITY DISTRIBUTIONS

Population centralization is measured by proxy using the dates as data approach[61–64]. The dates as data approach rests on the premise that larger and more centralized populations should produce and deposit greater amounts of datable materials relative to smaller and dispersed populations. This method has been increasingly refined and updated since its initial application in[62] and has been successfully used to track various aspects of population histories through time and across space[61,63–65].

The dates as data approach is nonetheless controversial, with several critiques emerging that question whether this method can accurately estimate population[66–68]. These critiques state that the dates as data approach actually tracks activity intensity or settlement aggregation rather than fluctuations in population[69–71]. Another issue is that fluctuations in the calibration curve can create artifacts in the data that look like changes in population[64,67,72]. To navigate these issues, we only use dates sampled from human skeletal material, the number of which is not affected by activity intensity. We also consider our population estimates to be a combination of population centralization and/or in-situ growth. Further, we use recent statistical improvements to the dates as data approach[73] that can assess whether variability in our population estimates are an artifact of sampling bias.

To estimate changes in population centralization at Mayapan we used the rcarbon package[73] in the R environment to generate summed probability distributions

(SPDs) on calibrated radiocarbon dates from our sample of 205 dated human skeletal remains. We present 25-, 50-, and 100-year smoothing parameter SPDs in Supplementary Figure 14, showing consistent centralization and decentralization timing. We follow the approach in[64] and compared our SPDs to a Monte-Carlo simulation of a null exponential growth model to identify general population trends as well as periods of time where our SPD significantly deviates from the expected null values. Generating the null exponential growth model consists of a three-stage process: 1) fitting a growth model to the observed SPD using a regression model; 2) generating random samples from the fitted model; and 3) un-calibrating the samples. The resulting set of radiocarbon dates can then be calibrated and aggregated in order to create an expected SPD of the fitted model that accounts for idiosyncrasies of the calibration process. Higher or lower than expected density of observed SPDs for a particular year will indicate a local divergence of the observed SPD from the fitted exponential growth null model, and the significance of these deviations can be used to assess the goodness-of-fit using a global test.

GENERALIZED LINEAR MODELS- BINOMIAL R^2 TERMS

Binomial R^2 terms are non-significant when comparing predictor variables to internal conflict due to the nature and structure of the response variable (Supplementary Table 2). Deaths from internal conflict is a binary variable that requires a binomial distribution be specified in the model that assumes a sigmoidal distribution. For the deaths from internal conflict to assume a sigmoidal distribution, and thus a significant R^2 term, the trend would require a shift from approximately zero deaths from internal conflict to 100% deaths across the x-axis. As this is exceedingly unlikely, the model fit will most often assume a linear trend that will lie on few if any observations, and thus residual variation will be very high (low R^2 term). We can see in Supplementary Figure 15 that while the R^2 terms are non-significant, deaths from civil conflict increase from ~2% when conditions are wettest to 20-60% when conditions are driest – certainly a significant effect. Due to the limitations inherent in the response data we include only the p-value term in the article text.

Supplementary Table 2 | Summary of quasibinomial GLM results. Significant p-values are in bold. Multivariate models include SPD as an interaction term to test whether climate effects internal conflict during periods of peak population centralization.

Model	Predictor	Response	p-value	Proportion Explained
Bivariate	YOK-I	Internal Conflict	**0.0033**	0.0633
Bivariate	Tzabnah	Internal Conflict	**0.0001**	0.1202
Bivariate	YOK-I	Population (SPD)	0.3030	0.0260
Bivariate	Tzabnah	Population (SPD)	0.3540	0.0340
Bivariate	SPD	Internal Conflict	0.4641	0.0037
Multivariate	YOK-I * SPD	Internal Conflict	0.4415	0.05817
Multivariate	Tzabnah * SPD	Internal Conflict	0.1754	0.10664

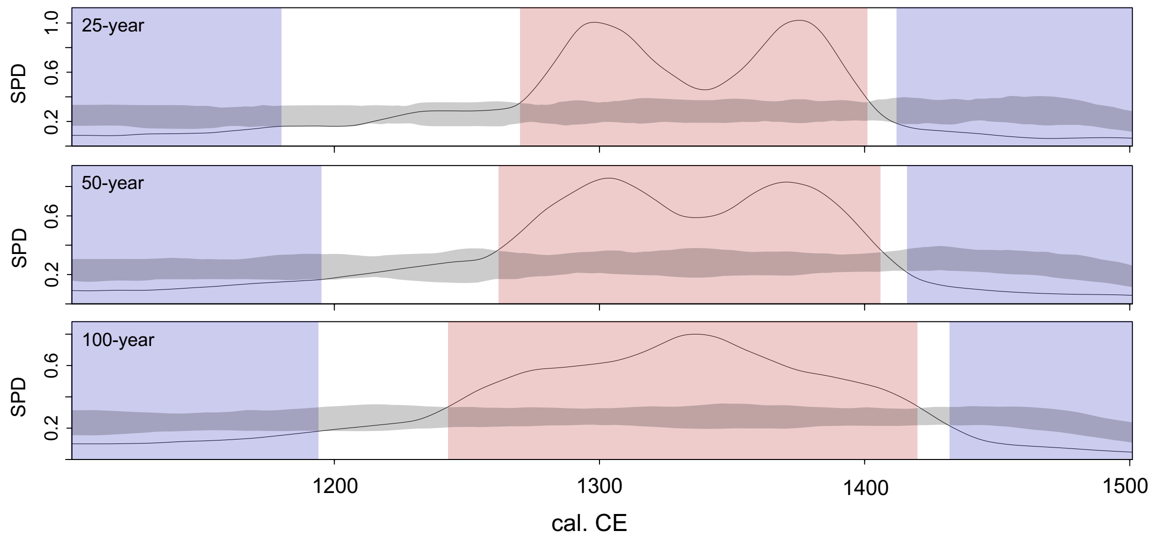

Supplementary Figure 14 | Mayapan summed probability distributions with three smoothing parameters: 25-year, 50-year, 100-year. All show population centralization in the latter half of the 13th century CE and decentralization beginning after 1350 cal. CE.

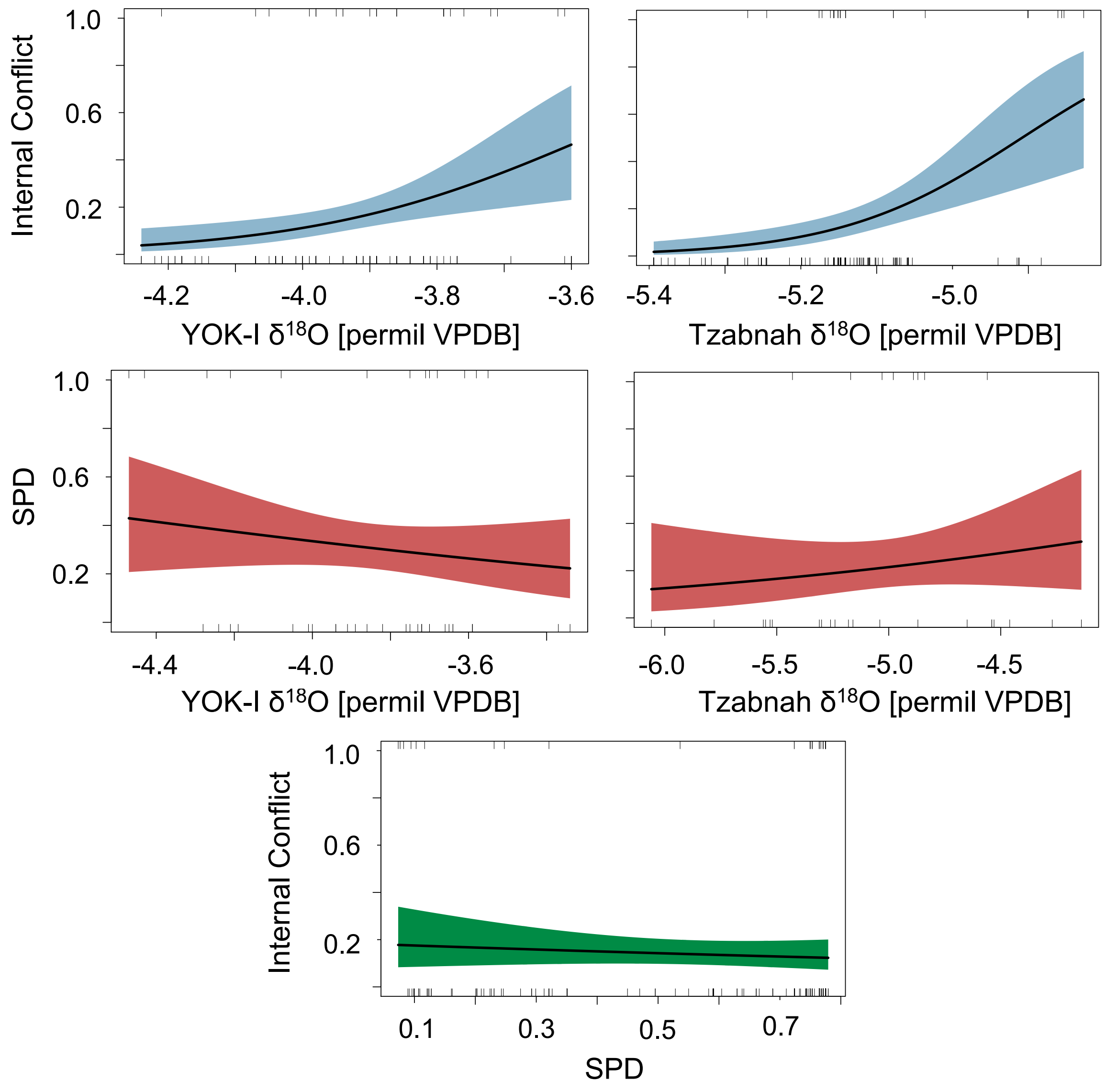

Supplementary Figure 15 | Bivariate Plots of GLMs. Plots of bivariate GLMs comparing deaths from internal conflict to YOK-I $\delta^{18}O$ and Tzabnah $\delta^{18}O$ in blue and the SPD (population estimate) in green. GLM comparing the population estimate (SPD) to YOK-I $\delta^{18}O$ and Tzabnah $\delta^{18}O$ are in red. Black ticks denote observations, bands show 95% confidence intervals.

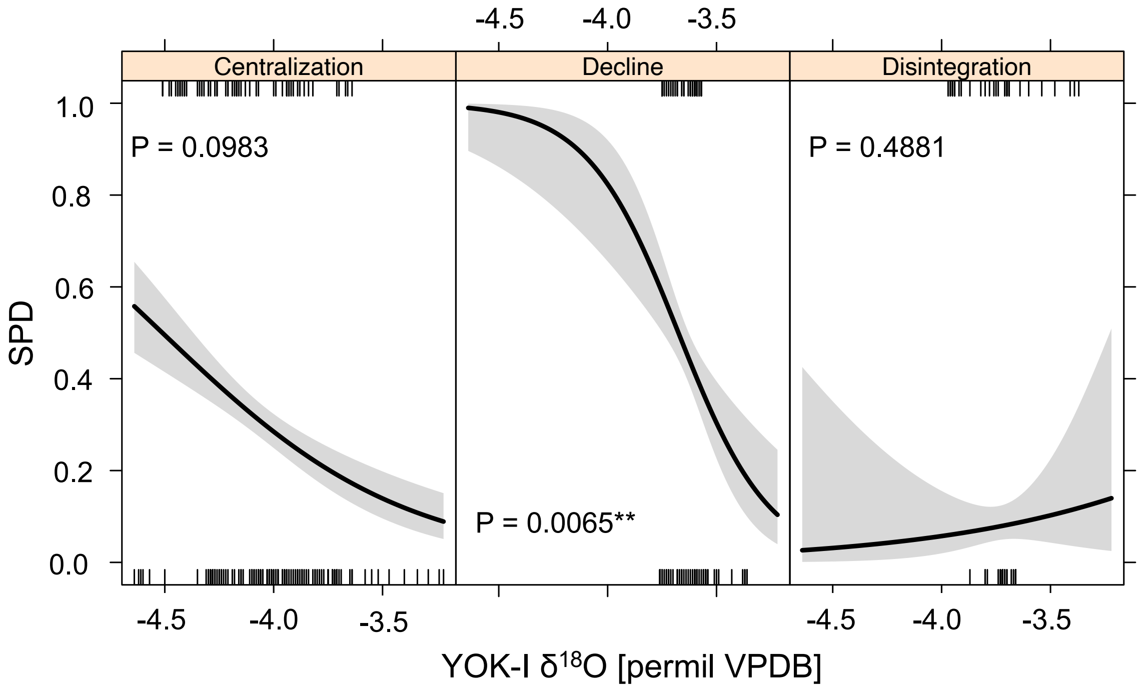

Supplementary Figure 16 | Population response to climate during centralization, decline and disintegration of Mayapan. Partial response plots of a multivariate GLM comparing SPD (population estimate) to YOK-I $\delta^{18}O$ with an interaction factor term breaking down the population trend into the centralization (<1350 cal. CE), decline (1350-1430 cal. CE) and disintegration (>1430 cal. CE) phases of Mayapan occupation. Black ticks denote observations, bands show 95% confidence intervals. The resolution of the local Tzabnah $\delta^{18}O$ climate record does not allow for this analysis.

Supplementary References

1. Kennett, D. J. *et al.* Development and disintegration of Maya political systems in response to climate change. *Science* **338**, 788–791 (2012).
2. Lechleitner, F. A. *et al.* Tropical rainfall over the last two millennia: evidence for a low-latitude hydrologic seesaw. *Scientific Reports* **7**, 1–9 (2017).
3. Smirnov, D. *et al.* A regime shift in the Sun-Climate connection with the end of the Medieval Climate Anomaly. *Scientific reports* **7**, 1–9 (2017).
4. Medina-Elizalde, M. *et al.* High resolution stalagmite climate record from the Yucatán Peninsula spanning the Maya terminal classic period. *Earth and Planetary Science Letters* **298**, 255–262 (2010).
5. Rehfeld, K., Marwan, N., Heitzig, J. & Kurths, J. Comparison of correlation analysis techniques for irregularly sampled time series. *Nonlinear Processes in Geophysics* **18**, 389–404 (2011).
6. Asmerom, Y. *et al.* Intertropical convergence zone variability in the Neotropics during the Common Era. *Science advances* **6**, eaax3644 (2020).
7. Breitenbach, S. F. *et al.* Constructing proxy records from age models (COPRA). *Climate of the Past* **8**, 1765–1779 (2012).
8. Hoggarth, J. A., Restall, M., Wood, J. W. & Kennett, D. J. Drought and Its Demographic Effects in the Maya Lowlands. *Current Anthropology* **58**, 82–113 (2017).
9. Brown, C. T. Caves, karst, and settlement at Mayapan, Yucatán. *In the maw of the earth monster: Mesoamerican ritual cave use* 373–402 (2005).
10. Brown, C. T. Water sources at Mayapan, Yucatán, Mexico, inPrecolumbian. *Water Management: Ideology, Ritual, and Politics* 171–85 (2006).
11. Weeks, J. M. & Masson, M. *The Carnegie Maya II: Carnegie Institution of Washington Current Reports, 1952-1957*. (University Press of Colorado, 2011).
12. Tan, M. *et al.* Applications of stalagmite laminae to paleoclimate reconstructions: comparison with dendrochronology/climatology. *Quaternary Science Reviews* **25**, 2103–2117 (2006).
13. Wassenburg, J. A. *et al.* Determination of aragonite trace element distribution coefficients from speleothem calcite–aragonite transitions. *Geochimica et Cosmochimica Acta* **190**, 347–367 (2016).
14. Lawrence Edwards, R., Chen, J. H. & Wasserburg, G. J. 238U234U230Th232Th systematics and the precise measurement of time over the past 500,000 years. *Earth and Planetary Science Letters* **81**, 175–192 (1987).
15. Frisia, S., Borsato, A., Fairchild, I. J., McDermott, F. & Selmo, E. M. Aragonite-calcite relationships in speleothems (Grotte de Clamouse, France): environment, fabrics, and carbonate geochemistry. *Journal of Sedimentary Research* **72**, 687–699 (2002).
16. Fohlmeister, J. *et al.* Carbon and oxygen isotope fractionation in the water-calcite-aragonite system. *Geochimica et Cosmochimica Acta* **235**, 127–139 (2018).
17. Lechleitner, F. A. *et al.* Hydrological and climatological controls on radiocarbon concentrations in a tropical stalagmite. *Geochimica et Cosmochimica Acta* **194**, 233–252 (2016).

18. Fohlmeister, J. *et al.* Main controls on the stable carbon isotope composition of speleothems. *Geochimica et Cosmochimica Acta* **279**, 67–87 (2020).
19. Hodell, D. A. *et al.* Climate change on the Yucatan Peninsula during the little ice age. *Quaternary Research* **63**, 109–121 (2005).
20. Reimer, P. *et al.* The IntCal20 Northern Hemisphere radiocarbon age calibration curve (0-55 kcal BP). *Radiocarbon* (2020).
21. Ramsey, C. B. Bayesian analysis of radiocarbon dates. *Radiocarbon* **51**, 337–360 (2009).
22. Ramsey, C. B. Deposition models for chronological records. *Quaternary Science Reviews* **27**, 42–60 (2008).
23. Ramsey, C. B. & Lee, S. Recent and planned developments of the program OxCal. *Radiocarbon* **55**, 720–730 (2013).
24. Paris, E. H. *et al.* Violence, desecration, and urban collapse at the Postclassic Maya political capital of Mayapan. *Journal of Anthropological Archaeology* **48**, 63–86 (2017).
25. Serafin, S. & Lope, C. P. Human sacrificial rites among the Maya of Mayapan: A bioarchaeological perspective. in *New perspectives on human sacrifice and ritual body treatments in ancient Maya society* 232–250 (Springer, 2007).
26. Serafin, S., Lope, C. P., González, E. U. & Kú, P. D. Odontometric investigation of the origin of freestanding shrine ossuaries at Mayapan. in *The Bioarchaeology of Space and Place* 141–167 (Springer, 2014).
27. Serafin, S., Lope, C. P. & Uc González, E. Bioarchaeological investigation of ancient Maya violence and warfare in inland northwest Yucatan, Mexico. *American journal of physical anthropology* **154**, 140–151 (2014).
28. Smith, A. L. Residential and associated structures at Mayapan. *Mayapan Yucatan, Mexico, Carnegie Institution of Washington Publication* (1962).
29. Serafin, S., González Uc & Delgado Kú. Prácticas Funerarias y Rituales en el Cenote San José de Mayapan. in *Prácticas Funerarias y Rituales en el Cenote San José de Mayapan* 81–91 (Archaeopress, 2014).
30. Hutchinson, R.H.H. A comparison of Mayapan's burial patterns within ritual buildings, in Settlement, Economy, and Society at Mayapan, Yucatan, Mexico. in *Settlement, Economy, and Society at Mayapan, Yucatan, Mexico* 183–206 (University of Pittsburgh Department of Anthropology, 2021).
31. Brown, C. T. *Mayapan society and ancient Maya social organization*. (Tulane University, 1999).
32. Mock, S. B. *The sowing and the dawning: termination, dedication, and transformation in the archaeological and ethnographic record of Mesoamerica*. (University of New Mexico Press, 1998).
33. Stanton, T. W., Brown, M. K. & Pagliaro, J. B. Garbage of the gods? Squatters, refuse disposal, and termination rituals among the ancient Maya. *Latin American Antiquity* **19**, 227–247 (2008).
34. Smith, M. E. City size in late postclassic Mesoamerica. *Journal of Urban History* **31**, 403–434 (2005).
35. Milbrath, S. & Peraza Lope, C. REVISITING MAYAPAN: Mexico's last Maya capital. *Ancient Mesoamerica* **14**, 1–46 (2003).

36. Peraza Lope, C. Mayapan, ciudad-capital del postclásico. *Arqueología Mexicana* **7**, 48–53 (1999).
37. Peraza Lope, C., Kú, P. D., Ojeda, B. E. & Euán, M. G. Trabajos de mantenimiento y conservación arquitectónica en Mayapan, Yucatán. Informe de la segunda temporada: 1997. *Instituto Nacional de Antropología e Historia, Merida, Yucatan, Mexico* (1999).
38. Shook, E. M. *Three temples and their associated structures at Mayapan*. (Carnegie Institution of Washington, Department of Archaeology, 1954).
39. Shook, E. M. & Irving, W. N. *Colonnaded Buildings at Mayapan*. (Carnegie Institution of Washington, Department of Archaeology, 1955).
40. Peraza Lope, C. *et al.* Trabajos de mantenimiento y conservación arquitectónica en Mayapan, Yucatán: Informe de la temporada 1996. *Centro INAH Yucatán* (1997).
41. Adams, R. M. *Some small ceremonial structures of Mayapan*. (Carnegie Institution of Washington, Department of Archaeology, 1953).
42. Masson, M. A. & Peraza Lope, C. *Kukulcan's Realm: Urban Life at Ancient Mayapan*. (University Press of Colorado, 2014).
43. Peraza Lope, C., Masson, M. A., Hare, T. S. & Kú, P. C. D. The chronology of Mayapan: New radiocarbon evidence. *Ancient Mesoamerica* **17**, 153–175 (2006).
44. Serafin, S. *Bioarchaeological investigation of violence at Mayapan*. (Tulane University, 2010).
45. Brown, C. T. New Views at Mayapan. in (First Annual Tulane Maya Symposium and Workshop, 2002).
46. Roys, R. L. Literary sources for the history of Mayapan. in *Mayapan, Yucatan, Mexico* 25–86 (Carnegie Institution of Washington, 1962).
47. Cen, A. H., Bastida, A. C., Tiesler, V. & Folan, W. J. Sacred spaces and human funerary and nonfunerary placements in Champotón, Campeche, during the Postclassic Period. in *New perspectives on human sacrifice and ritual body treatments in ancient Maya society* 209–231 (Springer, 2007).
48. Masson, M. A. & Peraza Lope, C. Kukulkan/Quetzalcoatl, death God, and creation mythology of burial shaft temples at Mayapan. *Mexicon* 77–85 (2007).
49. Peraza Lope, C., Delgado Kú, P. & Escamilla Ojeda, B. *Trabajos de mantenimiento y conservación arquitectónica en Mayapan, Yucatán. Informe de la tercera temporada: 1998. Informe de actividades al Consejo de Arqueología del Instituto Nacional de Antropología e Historia*. (2002).
50. Peraza Lope, Delgado Kú, P. & Escamilla Ojeda, B. *Trabajos de mantenimiento y conservación arquitectónica en Mayapan, Yucatán. Informe de la cuarta temporada: 1999-2000. Informe de actividades al Consejo de Arqueología del Instituto Nacional de Antropología e Historia*. (2003).
51. Paris, E. H. Metallurgy, Mayapan, and the Postclassic Mesoamerican world system. *Ancient Mesoamerica* **19**, 43–66 (2008).
52. Masson, M. A., Peraza Lope, C. & Hare, T. S. *Proyecto los Fundamentos del Poder Económico de Mayapan: Temporadas 2001–2004*. (2008).
53. Peraza Lope, C., Delgado Kú, P. & Escamilla Ojeda, B. Salvamento arqueológico en la modernización de la carretera Mérida-Mayapan-Oxkutzcab (Tramo Mayapan-Teabo). Segunda temporada: 1998. *Unpublished report submitted to the Instituto Nacional de Antropología e Historia, Mérida, Yucatan* (1998).

54. Masson, M. A., Peraza Lope, C., Hare, T. S. & Russell, B. W. *Proyecto los Fundamentos del Poder Económico de Mayapan: Temporadas 2008–2009*. (2012).
55. Smith, R. E. The place of Fine Orange pottery in Mesoamerican archaeology. *American Antiquity* **24**, 151–160 (1958).
56. Bishop, R. L. Pre-Columbian pottery: research in the Maya region. in 15–65 (1994).
57. Bishop, R. L. & Rands, R. L. Maya fine paste ceramics: a compositional perspective. in *Excavations at Seibal Memoirs* vol. 2 283–314 (1982).
58. Peraza Lope, C., Delgado Kú, P. & Escamilla Ojeda, B. *Trabajos de mantenimiento y conservación arquitectónica en Mayapan, Yucatán. Informe de la octava temporada: 2004-2005. Informe de actividades al Consejo de Arqueología del Instituto Nacional de Antropología e Historia*. (2005).
59. Peraza Lope, C., Delgado Kú, P. & Escamilla Ojeda, B. *Trabajos de mantenimiento y conservación arquitectónica en Mayapan, Yucatán. Informe de la novena temporada: 2005-2006. Informe de actividades al Consejo de Arqueología del Instituto Nacional de Antropología e Historia*. (2007).
60. Russell, B. W., Serafin, S., Uc González, E. & Peraza Lope, C. Underwater Investigations of Mass Burials in Two Cenotes at Mayapan, Yucatán, Mexico. in (2019).
61. Crema, E. R., Habu, J., Kobayashi, K. & Madella, M. Summed probability distribution of 14C dates suggests regional divergences in the population dynamics of the Jomon period in eastern Japan. *PLoS One* **11**, e0154809 (2016).
62. Rick, J. W. Dates as data: an examination of the Peruvian preceramic radiocarbon record. *American Antiquity* **52**, 55–73 (1987).
63. Broughton, J. M. & Weitzel, E. M. Population reconstructions for humans and megafauna suggest mixed causes for North American Pleistocene extinctions. *Nature communications* **9**, 1–12 (2018).
64. Shennan, S. *et al.* Regional population collapse followed initial agriculture booms in mid-Holocene Europe. *Nature communications* **4**, 1–8 (2013).
65. Bevan, A. *et al.* Holocene fluctuations in human population demonstrate repeated links to food production and climate. *Proceedings of the National Academy of Sciences* **114**, E10524–E10531 (2017).
66. Contreras, D. A. & Meadows, J. Summed radiocarbon calibrations as a population proxy: a critical evaluation using a realistic simulation approach. *Journal of Archaeological Science* **52**, 591–608 (2014).
67. Culleton, B. J. Crude demographic proxy reveals nothing about Paleoindian population. *Proceedings of the National Academy of Sciences* **105**, E111–E111 (2008).
68. Kennett, D. J., Stafford, T. W. & Southon, J. Standards of evidence and Paleoindian demographics. *Proceedings of the National Academy of Sciences* **105**, E107–E107 (2008).
69. Ebert, C. E., May, N. P., Culleton, B. J., Awe, J. J. & Kennett, D. J. Regional response to drought during the formation and decline of Preclassic Maya societies. *Quaternary Science Reviews* **173**, 211–235 (2017).
70. Hoggarth, J. A. *et al.* The political collapse of Chichen Itza in climatic and cultural context. *Global and planetary change* **138**, 25–42 (2016).

71. Kennett, D. J., Culleton, B. J., Dexter, J., Mensing, S. A. & Thomas, D. H. High-precision AMS 14C chronology for gatecliff shelter, Nevada. *Journal of Archaeological Science* **52**, 621–632 (2014).
72. Weninger, B., Clare, L., Jöris, O., Jung, R. & Edinborough, K. Quantum theory of radiocarbon calibration. *World Archaeology* **47**, 543–566 (2015).
73. Crema, E. R. & Bevan, A. Inference from large sets of radiocarbon dates: software and methods. *Radiocarbon* **63**, 23–39 (2021).
