# Supplementary material for: Drought-Induced Civil Conflict Among the Ancient Maya
Source: Nat Commun. 2022 Jul 19;13:3911. doi: 10.1038/s41467-022-31522-x (PMC9296624; doi:10.1038/s41467-022-31522-x)
Supplement: Supplementary file 3 — Description of Additional Supplementary Files [file 41467_2022_31522_MOESM3_ESM.pdf]

Description of Additional Supplementary Files

File Name: Supplementary Data 1
Description: Measured UTh isotope activity ratios and calculated U-Th ages.

File Name: Supplementary Data 2
Description: Osteological and chronological data.

File Name: Supplementary Code 1
Description: R markdown for SPD, phase differences in civil conflict, and regression models.

File Name: Supplementary Code 2
Description: OxCal Code used to model age of mass burials 1,2, 3 (shown in Figure 2b).
