# Supplementary material for: Drought-Induced Civil Conflict Among the Ancient Maya
Source: Nat Commun. 2022 Jul 19;13:3911. doi: 10.1038/s41467-022-31522-x (PMC9296624; doi:10.1038/s41467-022-31522-x)
Supplement: Supplementary file 8 — Reporting Summary [file 41467_2022_31522_MOESM8_ESM.pdf]

nature portfolio

Corresponding author(s): Douglas J. Kennett, David Hodell

Last updated by author(s): Jun 7, 2022

Reporting Summary

Nature Portfolio wishes to improve the reproducibility of the work that we publish. This form provides structure for consistency and transparency in reporting. For further information on Nature Portfolio policies, see our Editorial Policies and the Editorial Policy Checklist.

Statistics

For all statistical analyses, confirm that the following items are present in the figure legend, table legend, main text, or Methods section.

n/a	Confirmed	
☐	☒	The exact sample size (*n*) for each experimental group/condition, given as a discrete number and unit of measurement
☐	☒	A statement on whether measurements were taken from distinct samples or whether the same sample was measured repeatedly
☐	☒	The statistical test(s) used AND whether they are one- or two-sided<br>*Only common tests should be described solely by name; describe more complex techniques in the Methods section.*
☐	☒	A description of all covariates tested
☐	☒	A description of any assumptions or corrections, such as tests of normality and adjustment for multiple comparisons
☐	☒	A full description of the statistical parameters including central tendency (e.g. means) or other basic estimates (e.g. regression coefficient) AND variation (e.g. standard deviation) or associated estimates of uncertainty (e.g. confidence intervals)
☐	☒	For null hypothesis testing, the test statistic (e.g. *F*, *t*, *r*) with confidence intervals, effect sizes, degrees of freedom and *P* value noted<br>*Give P values as exact values whenever suitable.*
☐	☒	For Bayesian analysis, information on the choice of priors and Markov chain Monte Carlo settings
☒	☐	For hierarchical and complex designs, identification of the appropriate level for tests and full reporting of outcomes
☒	☐	Estimates of effect sizes (e.g. Cohen's *d*, Pearson's *r*), indicating how they were calculated

Our web collection on statistics for biologists contains articles on many of the points above.

Software and code

Policy information about availability of computer code

Data collection	Microsoft Excel version 16.6
Data analysis	Statistical analysis and SPD generation was conducted in the R programming environment version 1.4.1717 using the RCarbon package version 1.4.3. NESToolbox Version 1.01 was used to test if the spatially distant records were similar statistically and kernel-based correlation analysis. All dates were calibrated in OxCal version 4.462 using the IntCal20 curve.

For manuscripts utilizing custom algorithms or software that are central to the research but not yet described in published literature, software must be made available to editors and reviewers. We strongly encourage code deposition in a community repository (e.g. GitHub). See the Nature Portfolio guidelines for submitting code & software for further information.

Data

Policy information about availability of data

All manuscripts must include a data availability statement. This statement should provide the following information, where applicable:

- Accession codes, unique identifiers, or web links for publicly available datasets
- A description of any restrictions on data availability
- For clinical datasets or third party data, please ensure that the statement adheres to our policy

All relevant data are within the paper and its Supplementary Data files.

Field-specific reporting

Please select the one below that is the best fit for your research. If you are not sure, read the appropriate sections before making your selection.

- [ ] Life sciences
- [ ] Behavioural & social sciences
- [x] Ecological, evolutionary & environmental sciences

For a reference copy of the document with all sections, see nature.com/documents/nr-reporting-summary-flat.pdf

Ecological, evolutionary & environmental sciences study design

All studies must disclose on these points even when the disclosure is negative.

Study description

The influence of climate change on civil conflict and societal stability remains controversial, in part because of the limited number of case studies available for analysis that span the relatively stable climate conditions of the last century. We used archaeological, historical, and paleoclimate data to examine the relationship among climate change, civil conflict, and institutional collapse at Mayapán (1200-1450 cal. CE), the last large, Maya capital on the Yucatan Peninsula prior to European contact. We directly radiocarbon-dated 205 human skeletons from this city and combined these data with osteological analysis to evaluate the impact of climate change on civil conflict through time. The comparative paleoclimate record is derived from new local and existing regional speleothems and a sediment record from a nearby lake. We documented a 38% increase in violent deaths associated with drought conditions between 1400 and 1450 cal. CE. and use generalized linear modeling to demonstrate a direct causal link between drought and civil conflict. We conclude that prolonged drought aggravated factional tensions within the capital city's governing body that led to the fragmentation of the city by 1450 cal. CE.

Research sample

This interdisciplinary study utilizes directly radiocarbon dated human burials and osteological analysis (e.g., evidence for trauma) from multiple monumental and settlement burials at Mayapán and speleothem-based paleoclimate reconstructions. Burial selection was based on archaeological excavations across the urban center designed to obtain a representative and diverse sample from both the core and periphery of the urban population. This included 35 individuals < 18 years of age, 142 individuals ≥ 18 years of age (of which 48 are males, 49 are females and 45 are adults of indeterminate sex), and 28 individuals of unknown age.

The Mayapán speleothem Ml was collected from a cave below the city's central plaza approximately 20 m from the cave entrance. It represents the most local climate record possible. This record was compared to regional speleothem-based paleoclimate data from the Maya region, including the YOK-1 and YOK-G speleothem stable isotope records in Belize and an speleothem oxygen isotopic profile from Tzabnah Cave located 11.2 km north of Mayapán.

Sampling strategy

No sample-size calculation was performed for the number of individuals. Archaeological research in the last 70 years was designed to excavate different portions of the city starting with the city center and expanding out to different residential zones within the city wall. Burial selection was based on available archaeological contexts and the likelihood of successfully extracting datable material to develop the chronology, which effectively covered all regions of the site with recovered burials.

Data collection

M.M., C.P.L., B.W.R., E.U.G., and E.H.P. collected archaeological samples using standard methods; S.S. performed the osteological analysis using standardized methodologies; D.A.H., M.B., S.M., J.H.C. collected Mayapán speleothem and determined the U/Th age of samples using a Nu-Instrument Multicollector-Inductively-Coupled-Plasma-Mass Spectrometer (MC-ICP-MS); Fluorescent imaging was conducted using a Leica SP8 inverted confocal microscope; Layer counting has been repeated independently four times by two people (SFMB and SAC) counting twice each before comparing numbers; Stable O and C isotopes were measured using a Thermo Fisher Scientific Gasbench coupled to a Delta V isotope ratio mass spectrometer; D.J.K., B.J.C., R.J.G., J.A.H., and T.K.H. directly radiocarbon using a NEC 1.5SDH-1 Accelerator Costech elemental analyzer (ECS 4010) and a Thermo DeltaPlus Advantage isotope ratio mass spectrometer; D.A.H., S.F.M.B., K.M.P., T.C.S., N.M., M.Z., Y.A., V.J.P., V.V.A., S.A.C., D.H.J., A.J.M., G.H., M.B., J.U.L.B. compiled and analyzed published climate data.

Timing and spatial scale

Directly AMS 14C dated human burials indicate that the site was occupied as early as 700 cal. CE through 1450 cal. CE. Ancient individuals were sampled from laboratory collections recovered at Mayapán during field season directed by the Carnegie Institution of Washington (1950s), the Instituto Nacional de Antropología e Historia of Mexico (INAH; associated with the Proyecto Mayapán; 1996, 1997, 1998, 1999-2000, 2001, 2002, 2003, 2004-2005, 2007, 2009 the Salvamento Arqueológico en la Modernización de la Carretera Mérida-Mayapán-Oxkutzcab (1998) and the Salvamento Arqueológico Modernización de la Carretera Mérida-Chetumal (2015), the Proyecto Económico de Mayapán (PEMY; 2003, 2008, 2009, 2015), the Mayapán Taboo Cenote Project (1991).

In 2005, stalagmite M1 was retrieved from a karst cave located directly below the central plaza at Mayapán and accessible via Cenote Ch’en Mul.

Data exclusions

We assessed data quality via standard criteria for 14C AMS radiocarbon dates and excluded individuals with poor sample quality or evidence of contamination.

Reproducibility

AMS radiocarbon measurements result from an average of 10 sequential measurement and multiple burials were re-dated to verify the AMS radiocarbon date. All stable isotope data presented in the paper are average of 10 sequential measurements. All attempts to reproduce the presented data were successful.

Randomization

Analyses were conducted on individual burials from multiple site contexts and within subgroups (based demographic data, osteological data, and AMS radiocarbon dates) and compared to local and regional paleoclimate records.

Blinding

Analyses were performed similarly for all individuals.

Did the study involve field work? ☒ Yes ☐ No

Field work, collection and transport

Field conditions	Research projects at Mayapán have fostered cooperation between local and multi-national institutions since the late 19th century, and developed a rich breadth of archaeological and environmental data which has resulted in decades of published manuscripts and fueled ongoing field work. Mayapán field operations are located in the northern Yucatan Peninsula of Mexica. The environment is arid with low annual rainfall and temperatures typically ranging from 50-94 °F.
Location	The archaeological site of Mayapán is located in the Tecoh Municipality, Yucatan, Mexico (20.629444, -89.460556) at an elevation of ~52 feet.
Access & import/export	Permits were issued by the Instituto Nacional de Antropologfa e Historia (INAH) Consejo de Arqueologfa for excavation and the export of the samples for analysis and radiocarbon dating in the US (C.A.401-36/2172, issue date: October 21, 2009; 401-3-10492/11554, AA-53-09 A/ 3989, issue date: November 5, 2009; C.A.401-36/0028, issue date: January 19, 2010; C.A.401-36/1223, issue date: July 13, 2010; 401-3-7328, AA-40-10 A/ 948, issue date: August 10, 2010; 401-3-1016, AA-01-16, issue date: February 5, 2016).
Disturbance	All work was conducted on previously excavated archaeological materials rather than new excavations that would disturb the site further. We limited our sample of osteological material to approximately 100 mg to 1000 mg for each radiocarbon date to minimize disturbance to each burial. Considerations were made to judge the preservation of samples prior to analysis and limit the destruction of diagnostic features. The sampling strategies focused primarily on non-diagnostic skeletal elements and tooth roots to preserve the remaining material for future research projects.<br><br>To minimize impact we selected a single Mayapán speleothem MI based on field observations and carefully retrieved it from a karst cave located directly below the central plaza at Mayapán and accessible via Cenote Ch'en Mui.

Reporting for specific materials, systems and methods

We require information from authors about some types of materials, experimental systems and methods used in many studies. Here, indicate whether each material, system or method listed is relevant to your study. If you are not sure if a list item applies to your research, read the appropriate section before selecting a response.

Materials & experimental systems

n/a	Involved in the study
☒	☐ Antibodies
☒	☐ Eukaryotic cell lines
☐	☒ Palaeontology and archaeology
☒	☐ Animals and other organisms
☒	☐ Human research participants
☒	☐ Clinical data
☒	☐ Dual use research of concern

Methods

n/a	Involved in the study
☒	☐ ChIP-seq
☒	☐ Flow cytometry
☒	☐ MRI-based neuroimaging

Specimen provenance	Burials were excavated from a range of monumental and settlement context at Mayapán by the Carnegie Institution of Washington (CIW), the Instituto Nacional de Antropología e Historia of Mexico (INAH; associated with the Proyecto Mayapan, the Salvamento Arqueológico en la Modernización de la Carretera Merida-Mayapan-Oxkutzcab and the Salvamento Arqueológico Modernización de la Carretera Merida-Chetumal), the Proyecto Económico de Mayapán (PEMY), the Mayapán Taboo Cenote Project and as part of the dissertation research of Brown.<br><br>Permits were issued by the Instituto Nacional de Antropología e Historia (INAH) Consejo de Arqueológico for excavation and the export of samples for analysis in the US (C.A.401-36/2172, issue date: October 21, 2009; 401-3-10492/11554, AA-53-09 A/ 3989, issue date: November 5, 2009; C.A.401-36/0028, issue date: January 19, 2010; C.A.401-36/1223, issue date: July 13, 2010; 401-3-7328, AA-40-10 A/ 948, issue date: August 10, 2010; 401-3-1016, AA-01-16, issue date: February 5, 2016).
Specimen deposition	Human remains that fall under the auspices of the Instituto Nacional de Antropología e Historia of Mexico (Yucatan) are stored at a permanent repository located at the laboratory of the Proyecto INAH Mayapán, Telchaquillo, Yucatán, Mexico. Samples associated with the Carnegie excavations are stored at the laboratory of the Centro INAH Yucatán, Mérida, Yucatán, Mexico. All sample requests should be made to INAH.
Dating methods	Radiocarbon dates were obtained from 205 sets of human remains excavated at Mayapán and stored at the Proyecto INAH Mayapán in Telchaquillo, Mexico and the Centro INAH Yucatán, Mérida, Mexico. Bone collagen for radiocarbon analysis was processed at The Pennsylvania State University. Bone collagen was extracted and purified using the modified Longin method with ultrafiltration, with samples with low collagen yields were processed using amino acid hydrolysis and XAD purification. Physically cleaned samples were demineralized and gelatinized. Crude gelatin yields were recorded and the gelatin was ultrafiltered, retaining >30 kDa molecular weight gelatin. Carbon and nitrogen concentrations and stable isotope ratios were measured at the Yale Earth Systems Center for Stable Isotopic Studies facility with a Costech elemental analyzer (ECS 4010) and a Thermo Delta Plus Advantage isotope ratio mass spectrometer, respectively. Sample quality was evaluated by % crude gelatin yield, C%, N%, and C:N ratio. Ultrafiltered gelatin (~2.1 mg) was combusted for 3 hat 900 •c in vacuum-sealed quartz tubes with CuO and Ag wire. Graphitization and radiocarbon measurements were done at the Keck Carbon Cycle Accelerator Mass Spectrometer facility and the Penn State University Accelerator Mass Spectrometer laboratory. The radiocarbon ages were corrected for mass-dependent fractionation using measured carbon isotope values, and compared with backgrounds and known-age secondary standards. All dates were calibrated in OxCal version 4.462 using the IntCal20 curve.

☒ Tick this box to confirm that the raw and calibrated dates are available in the paper or in Supplementary Information.

Ethics oversight	Instituto Nacional de Antropología e Historia (INAH) Consejo de Arqueología for granting permits for excavation and the export of samples for analysis in the US (C.A.401-36/2172, issue date: October 21, 2009; 401-3-10492/11554, AA-53-09 A/ 3989, issue date: November 5, 2009; C.A.401-36/0028, issue date: January 19, 2010; C.A.401-36/1223, issue date: July 13, 2010; 401-3-7328, AA-40-10 A/ 948, issue date: August 10, 2010; 401-3-1016, AA-01-16, issue date: February 5, 2016).

Note that full information on the approval of the study protocol must also be provided in the manuscript.
