# Supplementary material for: Drought-Induced Civil Conflict Among the Ancient Maya
Source: Nat Commun. 2022 Jul 19;13:3911. doi: 10.1038/s41467-022-31522-x (PMC9296624; doi:10.1038/s41467-022-31522-x)
Supplement: Supplementary file 6 — Supplementary Code 1 [file 41467_2022_31522_MOESM6_ESM.pdf]

Kennett et al. Nature Comm.

Weston C. McCool

5/25/2022

R Markdown for SPD, phase differences in civil conflict, and regression models

Dataframes and packages

```
# Read in Mayapan dataframe
civil <- read.csv("./Dataframes/Conflict/phase and violence/Civil_Only.csv") # deaths from civil conflict
mayapan <- read.csv("./Dataframes/SPD/C14Data.csv") # uncalibrated direct bone dates
tzabnah_df <- read.csv("./Dataframes/Tzabnah/tzabnah_df.csv") # Tzabnah d18O values
yoki <-read.csv("./Dataframes/YOK-I/yoki_o18.csv") # YOK-I d18O values
spd <-read.csv("./Dataframes/SPD/mp_spd.csv") # SPD prob dens values
spd_tza_df <-read.csv("./Dataframes/SPD/tza_spd.csv") # DF with spd and Tzabnah values
spd_yoki_df <-read.csv("./Dataframes/SPD/yoki_spd.csv") # DF with spd and TOK-I values
spd_civil <-read.csv("./Dataframes/Conflict/civil_spd.csv") # DF with civil conflict and spd values
all_vars <-read.csv("./Dataframes/Conflict/civil_Tz_yok_spd.csv") # DF with civil, spd, YOK, Tza, values
SST <- read.csv("./Dataframes/Conflict/civil_sst.csv") # SST and civil conflict values

# Load packages
library(mgcv) # gams
```

```
## Loading required package: nlme
```

```
## This is mgcv 1.8-38. For overview type 'help("mgcv-package")'.
```

```
library(rcarbon) # SPDs
library(visreg) # plotting
library(modEvA) # D squared
```

SPD

```
mayapanC14 <- calibrate(x=mayapan$X14C_BP, errors=mayapan$Error, calCurves='intcal20', normalised=FALSE, verbose=
F) # calibrating mayapan dates
mayapanSPD = spd(mayapanC14, timeRange=c(1000, 350),runm=25) # un-normalized ranges 0-1
```

```
## [1] "Extracting and aggregating..."
## [1] "Done."
```

```
plot(mayapanSPD)
plot(mayapanSPD, runm=25, add=TRUE, type="simple", col="indianred", lwd=2, lty=2)
```

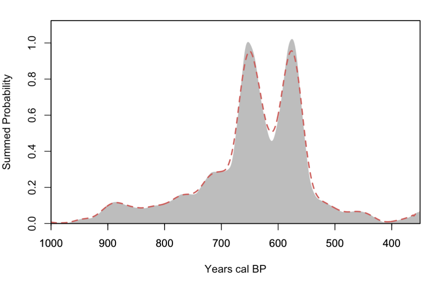


```
plot(mayapanSPD, calendar='BCAD', xlim=c(1100,1500))
```

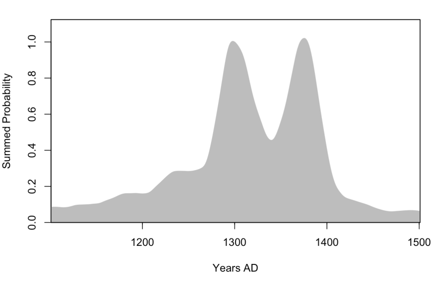


```
#### Generating the exponential growth null model via a Monte-Carlo simulation test. No. of simulations = 100
NullModel1 <- modelTest(mayapanC14, errors=mayapan$Error, nsim=100, timeRange=c(1000, 350), model="exponential",
runm=25, verbose=F) # 25-yr smooth
plot(NullModel1, calendar='BCAD', xlim=c(1100,1500), cex.lab=1.3, cex.axis=2, cex=, )
```

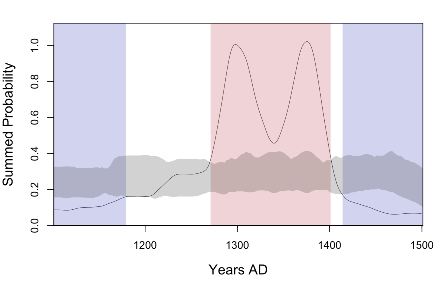


```
NullModel1$pval
```

```
## [1] 0.00990099
```

```
NullModel2 <- modelTest(mayapanC14, errors=mayapan$Error, nsim=100, timeRange=c(1000, 350), model="exponential",
runm=50, verbose=F) # 50-yr smooth
plot(NullModel2, calendar='BCAD', xlim=c(1100,1500), cex.lab=1.3, cex=100)
```

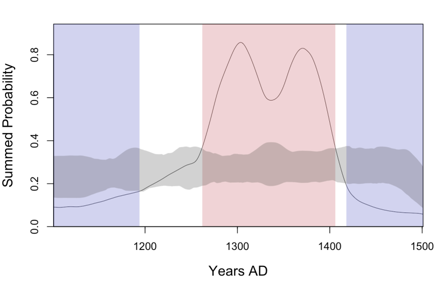


```
NullModel2$pval
```

```
## [1] 0.00990099
```

```
NullModel <- modelTest(mayapanC14, errors=mayapan$Error, nsim=100, timeRange=c(1000, 350), model="exponential", r
unm=100, verbose=F) # 100-yr smooth
plot(NullModel, calendar='BCAD', xlim=c(1100,1500))
```

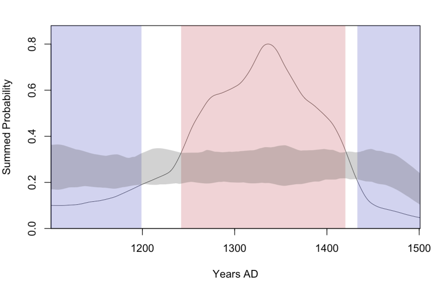


```
NullModel$pval
```

```
## [1] 0.00990099
```

Phase differences in civil conflict

```
# Civil Conflict
PhaseVD3 <- table(civil[, c("Period", "VD")])
PhaseVD3 <- as.data.frame.matrix(PhaseVD3)
# Early to Middle
table(x = PhaseVD3[c(1,4),])
```

```
##     No
## 1    66 74
##   4   1  0
##   13  0  1
```

```
EMP_MMP <- fisher.test(x = PhaseVD3[c(1,4),], alternative = "less")
EMP_MMP
```

```
##
##  Fisher's Exact Test for Count Data
##
## data:  PhaseVD3[c(1, 4), ]
## p-value = 0.05334
## alternative hypothesis: true odds ratio is less than 1
## 95 percent confidence interval:
##  0.000000 1.016515
## sample estimates:
## odds ratio
##  0.3471255
```

```
# Early to Late
table(x = PhaseVD3[c(1,3),])
```

```
##    No
## 1   8 66
##   4 0  1
##   9 1  0
```

```
EMP_LMP <- fisher.test(x = PhaseVD3[c(1,3),], alternative = "less")
EMP_LMP
```

```
##
##  Fisher's Exact Test for Count Data
##
## data:  PhaseVD3[c(1, 3), ]
## p-value = 2.285e-05
## alternative hypothesis: true odds ratio is less than 1
## 95 percent confidence interval:
##  0.0000000 0.2118785
## sample estimates:
## odds ratio
## 0.05715801
```

```
# Middle to Late
table(x = PhaseVD3[c(4,3),])
```

```
##     No
## 1    8 74
##   9  1  0
##   13 0  1
```

```
MMP_LMP <- fisher.test(x = PhaseVD3[c(4,3),], alternative = "less")
MMP_LMP
```

```
##
##  Fisher's Exact Test for Count Data
##
## data:  PhaseVD3[c(4, 3), ]
## p-value = 0.001489
## alternative hypothesis: true odds ratio is less than 1
## 95 percent confidence interval:
##  0.0000000 0.4689857
## sample estimates:
## odds ratio
##  0.1600884
```

Climate / Civil Conflict models

```
# GLM comparing civil conflict trauma to YOK-I averages for each individual
Civil_O18_2sd.glm <- glm(formula = Civil ~ Yok_O18,
                         data = all_vars,
                         family = quasibinomial(link = "logit"))
summary(Civil_O18_2sd.glm)
```

```
##
## Call:
## glm(formula = Civil ~ Yok_O18, family = quasibinomial(link = "logit"),
##     data = all_vars)
##
## Deviance Residuals:
##     Min       1Q   Median       3Q      Max
## -1.1179  -0.6030  -0.5099  -0.3211   2.5015
##
## Coefficients:
##             Estimate Std. Error t value Pr(>|t|)
## (Intercept)   17.222      6.324   2.723  0.00710 **
## Yok_O18        4.823      1.618   2.982  0.00326 **
## ---
## Signif. codes:  0 '***' 0.001 '**' 0.01 '*' 0.05 '.' 0.1 ' ' 1
##
## (Dispersion parameter for quasibinomial family taken to be 1.004348)
##
##     Null deviance: 153.14  on 182  degrees of freedom
## Residual deviance: 143.44  on 181  degrees of freedom
##   (28 observations deleted due to missingness)
## AIC: NA
##
## Number of Fisher Scoring iterations: 5
```

```
Dsquared(Civil_O18_2sd.glm)
```

```
## [1] 0.06332285
```

```
visreg(Civil_O18_2sd.glm, "Yok_O18", scale="response", partial=FALSE, line=list(col="black"),
       xlab="YOK-I δ18O [permil VPDB]", ylab="Internal Conflict",
       ylim=c(0.0,1.0), cex.axis=1.1, cex.lab=1.2, fill=list(col="lightskyblue3"))
```

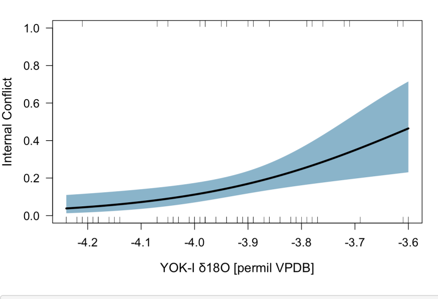


```
# GLM comparing civil conflict trauma to Tzabnah averages for each individual
Civil_TzO18_2sd.glm <- glm(formula = Civil ~ Tz_O18,
                         data = all_vars,
                         family = quasibinomial(link = "logit"))
summary(Civil_TzO18_2sd.glm)
```

```
##
## Call:
## glm(formula = Civil ~ Tz_O18, family = quasibinomial(link = "logit"),
##     data = all_vars)
##
## Deviance Residuals:
##     Min       1Q   Median       3Q      Max
## -1.2682  -0.5516  -0.4890  -0.2934   2.4698
##
## Coefficients:
##             Estimate Std. Error t value Pr(>|t|)
## (Intercept)   40.742     10.703   3.806 0.000193 ***
## Tz_O18         8.300      2.099   3.954 0.000110 ***
## ---
## Signif. codes:  0 '***' 0.001 '**' 0.01 '*' 0.05 '.' 0.1 ' ' 1
##
## (Dispersion parameter for quasibinomial family taken to be 1.035342)
##
##     Null deviance: 153.14  on 182  degrees of freedom
## Residual deviance: 134.75  on 181  degrees of freedom
##   (28 observations deleted due to missingness)
## AIC: NA
##
## Number of Fisher Scoring iterations: 5
```

```
Dsquared(Civil_TzO18_2sd.glm)
```

```
## [1] 0.1201071
```

```
visreg(Civil_TzO18_2sd.glm, "Tz_O18", scale="response", partial=FALSE, line=list(col="black"),
       xlab="Tzabnah δ18O [permil VPDB]", ylab="Internal Conflict",
       ylim=c(0.0,1.0), cex.axis=1.1, cex.lab=1.2, fill=list(col="lightskyblue3"))
```

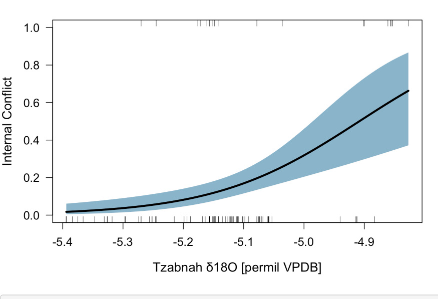


```
## GLM comparing civil conflict to  Cariaco average SST summer/fall temps averages for each individual
sstSF.glm <- glm(formula = Civil ~ sst_sf,
                 data = SST,
                 family = quasibinomial)
summary(sstSF.glm)
```

```
##
## Call:
## glm(formula = Civil ~ sst_sf, family = quasibinomial, data = SST)
##
## Deviance Residuals:
##     Min       1Q   Median       3Q      Max
## -0.5965  -0.5794  -0.5779  -0.5207   2.0221
##
## Coefficients:
##             Estimate Std. Error t value Pr(>|t|)
## (Intercept)   14.630     35.813   0.409    0.683
## sst_sf        -0.577      1.262  -0.457    0.648
##
## (Dispersion parameter for quasibinomial family taken to be 1.009722)
##
##     Null deviance: 153.14  on 182  degrees of freedom
## Residual deviance: 152.92  on 181  degrees of freedom
##   (28 observations deleted due to missingness)
## AIC: NA
##
## Number of Fisher Scoring iterations: 4
```

```
Dsquared(model = sstSF.glm)
```

```
## [1] 0.001430836
```

Demography / Climate models

```
# GLM comparing SPD to YOK-I values
spd_yok1 <- glm(spd ~ d18O,
               data=spd_yoki_df[c(TRUE, rep(FALSE, 15)),], # 15 year re-sample to reduce temporal autocorr.
               family=quasibinomial(link = "logit"))
summary(spd_yok1)
```

```
##
## Call:
## glm(formula = spd ~ d18O, family = quasibinomial(link = "logit"),
##     data = spd_yoki_df[c(TRUE, rep(FALSE, 15)), ])
##
## Deviance Residuals:
##     Min       1Q   Median       3Q      Max
## -0.6681  -0.4638  -0.3256   0.1683   1.5946
##
## Coefficients:
##             Estimate Std. Error t value Pr(>|t|)
## (Intercept)  -4.1038     3.1761  -1.292    0.204
## d18O         -0.8545     0.8185  -1.044    0.303
##
## (Dispersion parameter for quasibinomial family taken to be 0.4314869)
##
##     Null deviance: 17.905  on 40  degrees of freedom
## Residual deviance: 17.435  on 39  degrees of freedom
##   (17 observations deleted due to missingness)
## AIC: NA
##
## Number of Fisher Scoring iterations: 3
```

```
acf(x=resid(spd_yok1), main=NA)
```

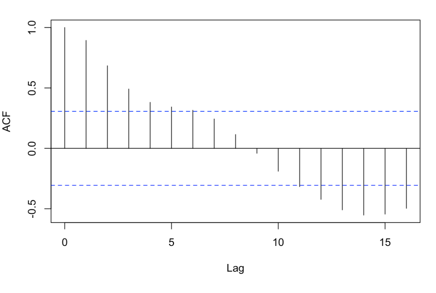


```
Dsquared(model = spd_yok1)
```

```
## [1] 0.02628305
```

```
visreg(spd_yok1, "d18O", scale="response", partial=FALSE, line=list(col="black"),
       xlab="Yok-I δ18O", ylab="SPD", ylim=c(0.0,1.0),
       cex.axis=1.1, cex.lab=1.2, fill=list(col="indianred"))
```

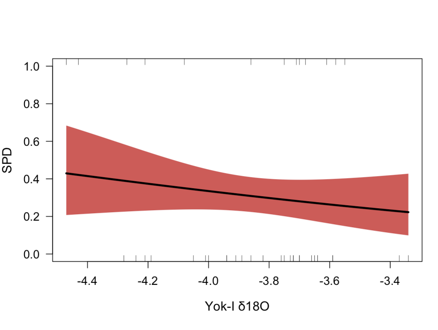


```
# A GAM to assess whether a non-linear model performs better
spd_yok2 <- gam(spd ~ s(d18O),
               data=spd_yoki_df,
               family=quasibinomial(link = "logit"))
summary(spd_yok2)
```

```
##
## Family: quasibinomial
## Link function: logit
##
## Formula:
## spd ~ s(d18O)
##
## Parametric coefficients:
##             Estimate Std. Error t value Pr(>|t|)
## (Intercept) -0.86229    0.05343  -16.14   <2e-16 ***
## ---
## Signif. codes:  0 '***' 0.001 '**' 0.01 '*' 0.05 '.' 0.1 ' ' 1
##
## Approximate significance of smooth terms:
##           edf Ref.df     F p-value
## s(d18O) 8.784  8.985 8.367  <2e-16 ***
## ---
## Signif. codes:  0 '***' 0.001 '**' 0.01 '*' 0.05 '.' 0.1 ' ' 1
##
## R-sq.(adj) =  0.119   Deviance explained = 11.9%
## GCV = 0.38474  Scale est. = 0.36894   n = 674
```

```
plot(spd_yok2) # trend is very inconsistent, need to hold knots to linear, so back to GLM
```

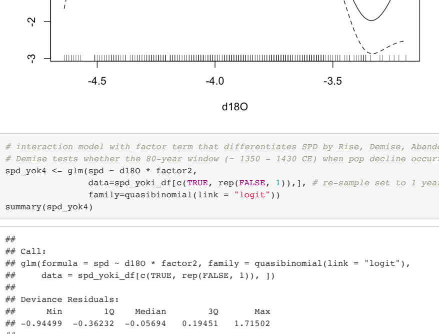


```
# interaction model with factor term that differentiates SPD by Rise, Demise, Abandonment
# Demise tests whether the 80-year window (~ 1350 - 1430 CE) when pop decline occurred coincides with drought
spd_yok4 <- glm(spd ~ d18O * factor2,
               data=spd_yoki_df[c(TRUE, rep(FALSE, 1)),], # re-sample set to 1 year
               family=quasibinomial(link = "logit"))
summary(spd_yok4)
```

```
##
## Call:
## glm(formula = spd ~ d18O * factor2, family = quasibinomial(link = "logit"),
##     data = spd_yoki_df[c(TRUE, rep(FALSE, 1)), ])
##
## Deviance Residuals:
##      Min        1Q    Median        3Q       Max
## -0.94499  -0.36232  -0.05694   0.19451   1.71502
##
## Coefficients:
##                     Estimate Std. Error t value Pr(>|t|)
## (Intercept)            2.243      6.766   0.332  0.74046
## d18O                   1.261      1.817   0.694  0.48808
## factor2Demise        -19.668      8.111  -2.425  0.01584 *
## factor2Rise          -10.372      6.906  -1.502  0.13405
## d18O:factor2Demise    -6.003      2.193  -2.737  0.00654 **
## d18O:factor2Rise      -3.063      1.848  -1.658  0.09834 .
## ---
## Signif. codes:  0 '***' 0.001 '**' 0.01 '*' 0.05 '.' 0.1 ' ' 1
##
## (Dispersion parameter for quasibinomial family taken to be 0.2862938)
##
##     Null deviance: 143.00  on 336  degrees of freedom
## Residual deviance: 101.53  on 331  degrees of freedom
##   (126 observations deleted due to missingness)
## AIC: NA
##
## Number of Fisher Scoring iterations: 5
```

```
Dsquared(model = spd_yok4)
```

```
## [1] 0.2899929
```

```
v<- visreg(spd_yok4, "d18O", by="factor2", scale="response", partial=FALSE,
          layout=c(3,1), line=list(col="black"),
          xlab="YOK-I d18O [permil VPDB]", ylab="SPD")
```

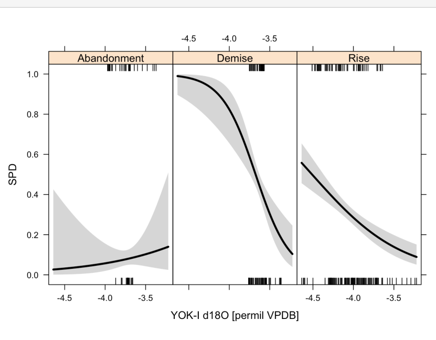


```
acf(x=resid(spd_yok4), main=NA) # there is TAC
```

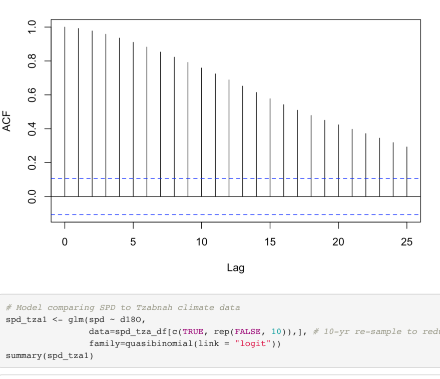


```
# Model comparing SPD to Tzabnah climate data
spd_tza1 <- glm(spd ~ d18O,
               data=spd_tza_df[c(TRUE, rep(FALSE, 10)),], # 10-yr re-sample to reduce TAC
               family=quasibinomial(link = "logit"))
summary(spd_tza1)
```

```
##
## Call:
## glm(formula = spd ~ d18O, family = quasibinomial(link = "logit"),
##     data = spd_tza_df[c(TRUE, rep(FALSE, 10)), ])
##
## Deviance Residuals:
##      Min        1Q    Median        3Q       Max
## -0.71443  -0.48090  -0.34698   0.01267   1.71107
##
## Coefficients:
##             Estimate Std. Error t value Pr(>|t|)
## (Intercept)  -2.8355     5.3639  -0.529    0.606
## d18O         -0.3097     1.0444  -0.297    0.771
##
## (Dispersion parameter for quasibinomial family taken to be 0.5219191)
##
##     Null deviance: 6.4027  on 14  degrees of freedom
## Residual deviance: 6.3568  on 13  degrees of freedom
##   (45 observations deleted due to missingness)
## AIC: NA
##
## Number of Fisher Scoring iterations: 4
```

```
acf(x=resid(spd_tza1), main=NA)
```

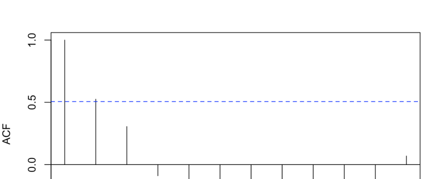

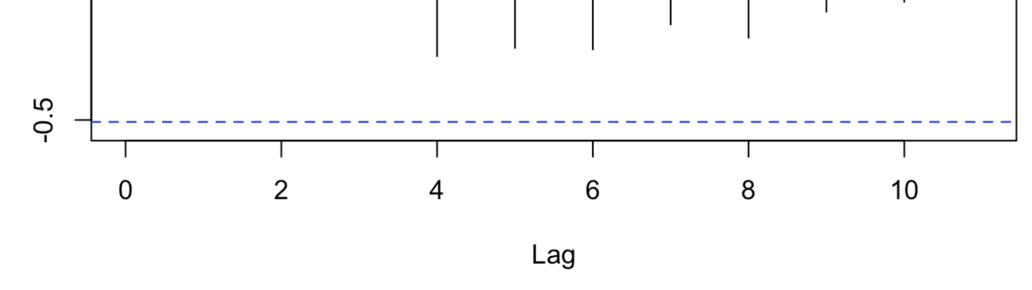


```
Dsquared(model = spd_tza1)
```

```
## [1] 0.007173004
```

```
visreg(spd_tza1, "d18O", scale="response", partial=FALSE, line=list(col="black"),
       xlab="Tzabnah δ18O", ylab="SPD", ylim=c(0.0,1.0),
       cex.axis=1.1, cex.lab=1.2, fill=list(col="indianred"))
```

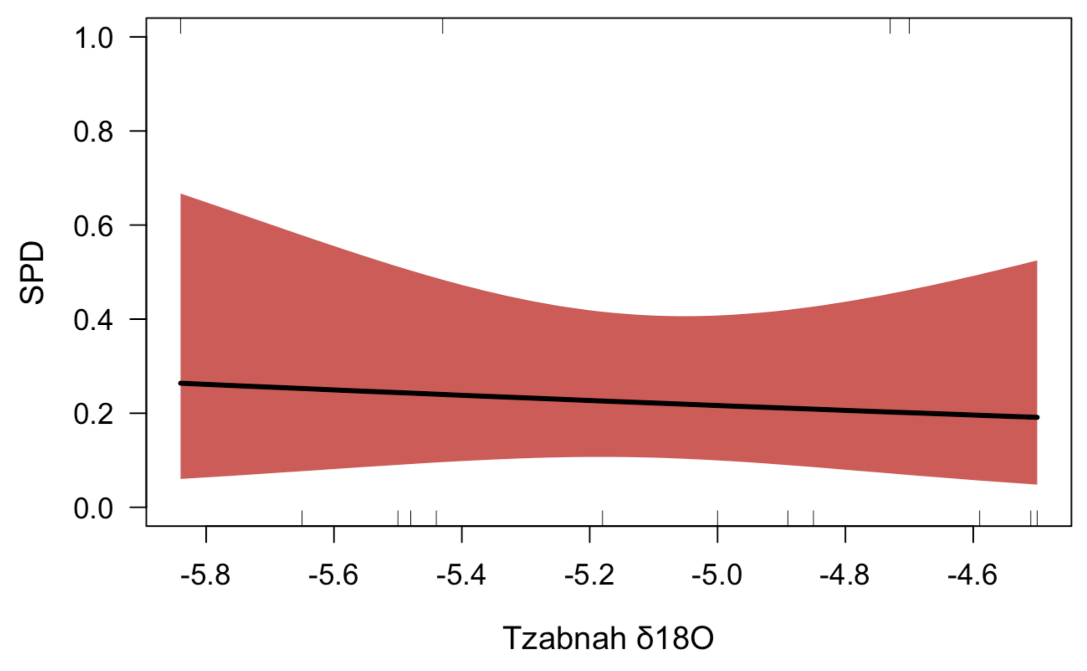


```
# How does a GAM perform?
spd_tza1.1 <- gam(spd ~ s(d18O),
                data=spd_tza_df[c(TRUE, rep(FALSE, 10)),],
                family=quasibinomial(link = "logit"))
summary(spd_tza1.1)
```

```
##
## Family: quasibinomial
## Link function: logit
##
## Formula:
## spd ~ s(d18O)
##
## Parametric coefficients:
##             Estimate Std. Error t value Pr(>|t|)
## (Intercept)  -1.2602     0.4507  -2.796   0.0152 *
## ---
## Signif. codes:  0 '***' 0.001 '**' 0.01 '*' 0.05 '.' 0.1 ' ' 1
##
## Approximate significance of smooth terms:
##         edf Ref.df     F p-value
## s(d18O)   1      1 0.088   0.771
##
## R-sq.(adj) =  -0.0688   Deviance explained = 0.717%
## GCV = 0.56422  Scale est. = 0.52153   n = 15
```

SPD / Civil Conflict models

```
spd_civil1 <- glm(Civil ~ SPD,
                data=spd_civil,
                family=quasibinomial(link = "logit"))
summary(spd_civil1)
```

```
##
## Call:
## glm(formula = Civil ~ SPD, family = quasibinomial(link = "logit"),
##     data = spd_civil)
##
## Deviance Residuals:
##     Min       1Q   Median       3Q      Max
## -0.6225  -0.5668  -0.5176  -0.5138   2.0457
##
## Coefficients:
##             Estimate Std. Error t value Pr(>|t|)
## (Intercept)  -1.4884     0.4964  -2.998   0.0031 **
## SPD          -0.6097     0.8310  -0.734   0.4641
## ---
## Signif. codes:  0 '***' 0.001 '**' 0.01 '*' 0.05 '.' 0.1 ' ' 1
##
## (Dispersion parameter for quasibinomial family taken to be 1.013755)
##
##     Null deviance: 145.06  on 179  degrees of freedom
## Residual deviance: 144.53  on 178  degrees of freedom
##   (31 observations deleted due to missingness)
## AIC: NA
##
## Number of Fisher Scoring iterations: 4
```

```
acf(x=resid(spd_civil1), main=NA) #  there is TAC
```

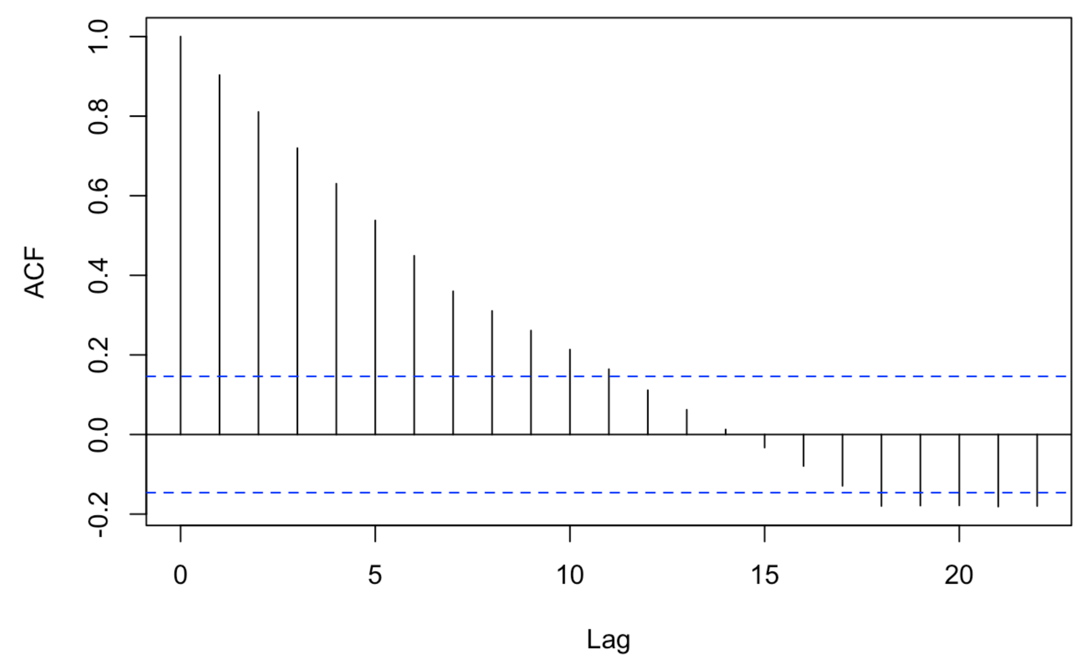


```
Dsquared(model = spd_civil1)
```

```
## [1] 0.00367965
```

```
visreg(spd_civil1, "SPD", scale="response", partial=FALSE, line=list(col="black"),
       xlab="SPD", ylab="Internal Conflict", ylim=c(0.0,1.0),
       cex.axis=1.1, cex.lab=1.2, fill=list(col="springgreen4"))
```

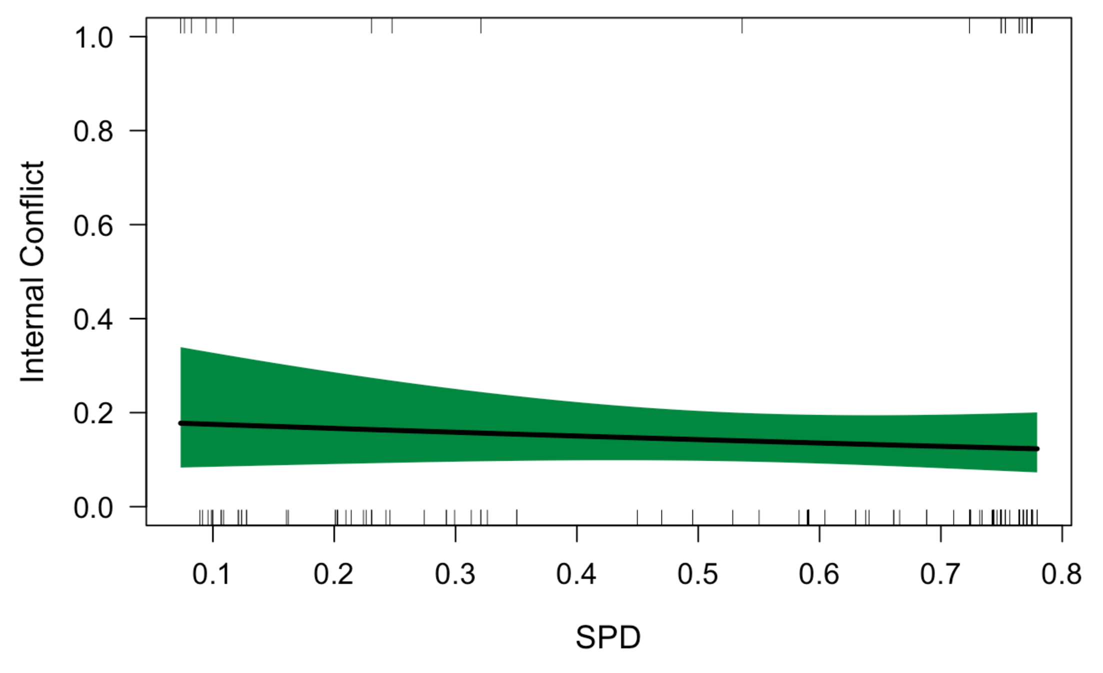


```
# does a GAM perform better?
spd_civil1.1 <- gam(Civil ~ s(SPD),
                data=spd_civil,
                family=quasibinomial(link = "logit"))
summary(spd_civil1.1)
```

```
##
## Family: quasibinomial
## Link function: logit
##
## Formula:
## Civil ~ s(SPD)
##
## Parametric coefficients:
##             Estimate Std. Error t value Pr(>|t|)
## (Intercept)  -1.9187     0.2346  -8.179 5.43e-14 ***
## ---
## Signif. codes:  0 '***' 0.001 '**' 0.01 '*' 0.05 '.' 0.1 ' ' 1
##
## Approximate significance of smooth terms:
##          edf Ref.df     F p-value
## s(SPD) 2.459  3.013 1.943   0.124
##
## R-sq.(adj) =  0.0375   Deviance explained = 5.29%
## GCV = 0.79345  Scale est. = 1.0196    n = 180
```

Multivariate models

```
# Does drought (Yok-I) only increase civil violence when pop density is high?
inter1 <- glm(Civil ~ Yok_O18 * SPD,
                  data=all_vars,
                  family=quasibinomial(link = "logit"))
summary(inter1)
```

```
##
## Call:
## glm(formula = Civil ~ Yok_O18 * SPD, family = quasibinomial(link = "logit"),
##     data = all_vars)
##
## Deviance Residuals:
##     Min       1Q   Median       3Q      Max
## -1.1992  -0.5593  -0.5208  -0.3885   2.5035
##
## Coefficients:
##             Estimate Std. Error t value Pr(>|t|)
## (Intercept)   23.497     12.703   1.850   0.0660 .
## Yok_O18        6.444      3.303   1.951   0.0527 .
## SPD          -24.039     30.906  -0.778   0.4377
## Yok_O18:SPD   -6.113      7.925  -0.771   0.4415
## ---
## Signif. codes:  0 '***' 0.001 '**' 0.01 '*' 0.05 '.' 0.1 ' ' 1
##
## (Dispersion parameter for quasibinomial family taken to be 1.032797)
##
##     Null deviance: 145.06  on 179  degrees of freedom
## Residual deviance: 136.62  on 176  degrees of freedom
##   (31 observations deleted due to missingness)
## AIC: NA
##
## Number of Fisher Scoring iterations: 5
```

```
# # Does drought (Tzabnah) only increase civil violence when pop density is high?
inter2 <- glm(Civil ~ Tz_O18 * SPD,
              data=all_vars,
              family=quasibinomial(link = "logit"))
summary(inter2)
```

```
##
## Call:
## glm(formula = Civil ~ Tz_O18 * SPD, family = quasibinomial(link = "logit"),
##     data = all_vars)
##
## Deviance Residuals:
##     Min       1Q   Median       3Q      Max
## -1.2184  -0.5553  -0.5447  -0.2800   2.6711
##
## Coefficients:
##             Estimate Std. Error t value Pr(>|t|)
## (Intercept)   65.353     24.515   2.666  0.00840 **
## Tz_O18        13.271      4.896   2.711  0.00738 **
## SPD          -82.359     60.987  -1.350  0.17861
## Tz_O18:SPD   -16.295     11.978  -1.360  0.17543
## ---
## Signif. codes:  0 '***' 0.001 '**' 0.01 '*' 0.05 '.' 0.1 ' ' 1
##
## (Dispersion parameter for quasibinomial family taken to be 1.170172)
##
##     Null deviance: 145.06  on 179  degrees of freedom
## Residual deviance: 129.59  on 176  degrees of freedom
##   (31 observations deleted due to missingness)
## AIC: NA
##
## Number of Fisher Scoring iterations: 5
```
